# Remarkable agrivoltaic influence on soil moisture, micrometeorology and water-use efficiency

**DOI:** 10.1371/journal.pone.0203256

**Published:** 2018-11-01

**Authors:** Elnaz Hassanpour Adeh, John S. Selker, Chad W. Higgins

**Affiliations:** Department of Biological and Ecological Engineering, Oregon State University, Corvallis, Oregon, United States of America; Universita degli Studi della Tuscia, ITALY

## Abstract

Power demands are set to increase by two-fold within the current century and a high fraction of that demand should be met by carbon free sources. Among the renewable energies, solar energy is among the fastest growing; therefore, a comprehensive and accurate design methodology for solar systems and how they interact with the local environment is vital. This paper addresses the environmental effects of solar panels on an unirrigated pasture that often experiences water stress. Changes to the microclimatology, soil moisture, water usage, and biomass productivity due to the presence of solar panels were quantified. The goal of this study was to show that the impacts of these factors should be considered in designing the solar farms to take advantage of potential net gains in agricultural and power production. Microclimatological stations were placed in the Rabbit Hills agrivoltaic solar arrays, located in Oregon State campus, two years after the solar array was installed. Soil moisture was quantified using neutron probe readings. Significant differences in mean air temperature, relative humidity, wind speed, wind direction, and soil moisture were observed. Areas under PV solar panels maintained higher soil moisture throughout the period of observation. A significant increase in late season biomass was also observed for areas under the PV panels (90% more biomass), and areas under PV panels were significantly more water efficient (328% more efficient).

## 1 Introduction

Global energy demand will be doubled by mid-century due to population and economic growth [1,2]. Renewable and environmental-friendly energies will play a vital role to meet this demand.

Among all renewable energies, solar power is the most abundant and available source [3]. Solar power is also becoming more affordable. The cost of solar panels has fallen by 10% per year for the past thirty years, while production has risen by 30% per year. If costs continue to be reduced based on this historic rate, solar energy will be less expensive than coal by 2020[4]. The impact of wide-spread solar installations is an area of increasing interest. Regional climatology may be influenced by large scale solar installations, but simulations have provided conflicting results: 3–4°C *increase* in air temperature over solar panels compared to wildlands at night [5], 0.1–0.5°C *decrease* in air temperature [6], 26°C *increase* in the shaded roof top temperature compared with unshaded roof top [7], 1–2.5°C increase in regional and global temperatures in urban area [8] and a 5.2°C *increase* in air temperature under solar panels [9].

Solar installations can occupy large land areas and sometimes compete with agriculture for the land resource [10]. Agrivoltaic systems are created when solar and agricultural systems are co-located for mutual benefit. The formal introduction of agrivoltaic systems is credited to Dupraz in 2011 [11]. Land demand for energy production decreases profoundly when agrivoltaics are used [10]. Not all agricultural crops are suitable, but plants with less root density and a high net photosynthetic rate are ideal candidates [11]. Agrivoltaic systems have been shown to increase land productivity by 60–70% [12], and increase the value of energy production system by 30% [13]. Very limited experimental research was found on the impacts of a solar arrays on agricultural production. Marrou et al. [14] measured soil water potential and soil water gradient (difference between uptake and drainage) in cucumber and lettuce and revealed lower soil water potential under the panels. This water potential led to an increase in harvested final fresh weight. Another experiment by Marrou et el. [15] found that plants cover soil faster under the shade of solar panels. An experimental study by Dupraz et al. demonstrated that summer crops benefited of solar shade more than winter crops such as pea and wheat crops [16]. Co-locating agave plant below solar panels increased yield per m^3^ of water used in the San Bernardino County in California [17]. Non-beneficial effects have also been observed in Welch onion fields where, photovoltaics reduced the fresh and dry matter harvest weight [18].

In this paper, a field study was performed to measure the effects of a six-acre agrivoltaic solar farm on the microclimatology, soil moisture and pasture production. The experimental setup included microclimatological and soil moisture measurements from May to August 2015 in Rabbit Hills agrivoltaic solar arrays, located on the Oregon State University campus. The field data for this study is accessible through Oregon State library system [19].

## 2 Material and methods

**Fig 1:**
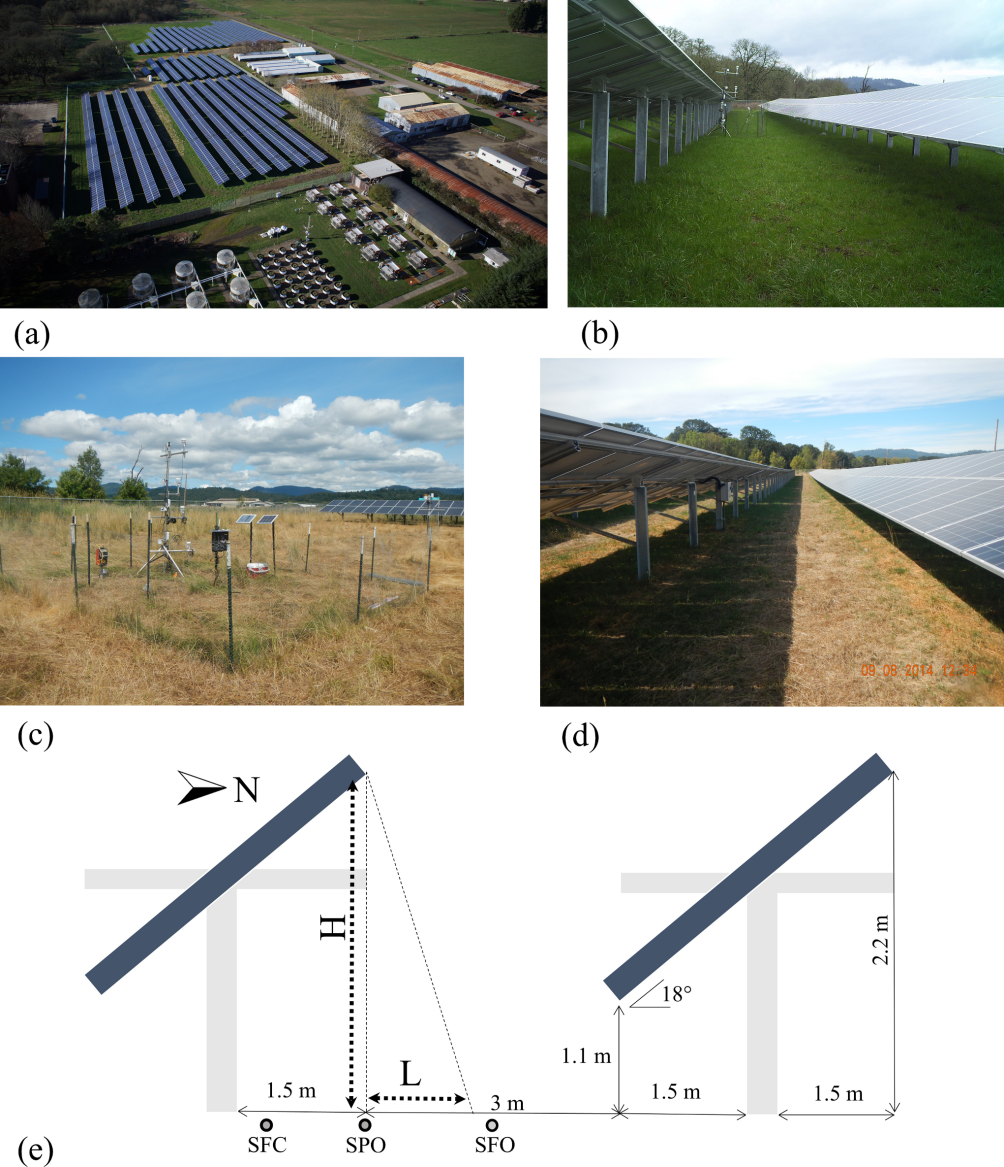
a) Aerial photo of 35^th^ Street agrivoltaic solar array, Oregon State University Corvallis campus (this photo is taken in winter and shadow pattern is different from the measurements which held in summer) Copyright: Oregon State University, b) Solar panel set up, c) Control area set up, d) Shade zones in solar panel, e) Schematic drawing of shade zones (H is object height and L is shadow length).

**Fig 2:**
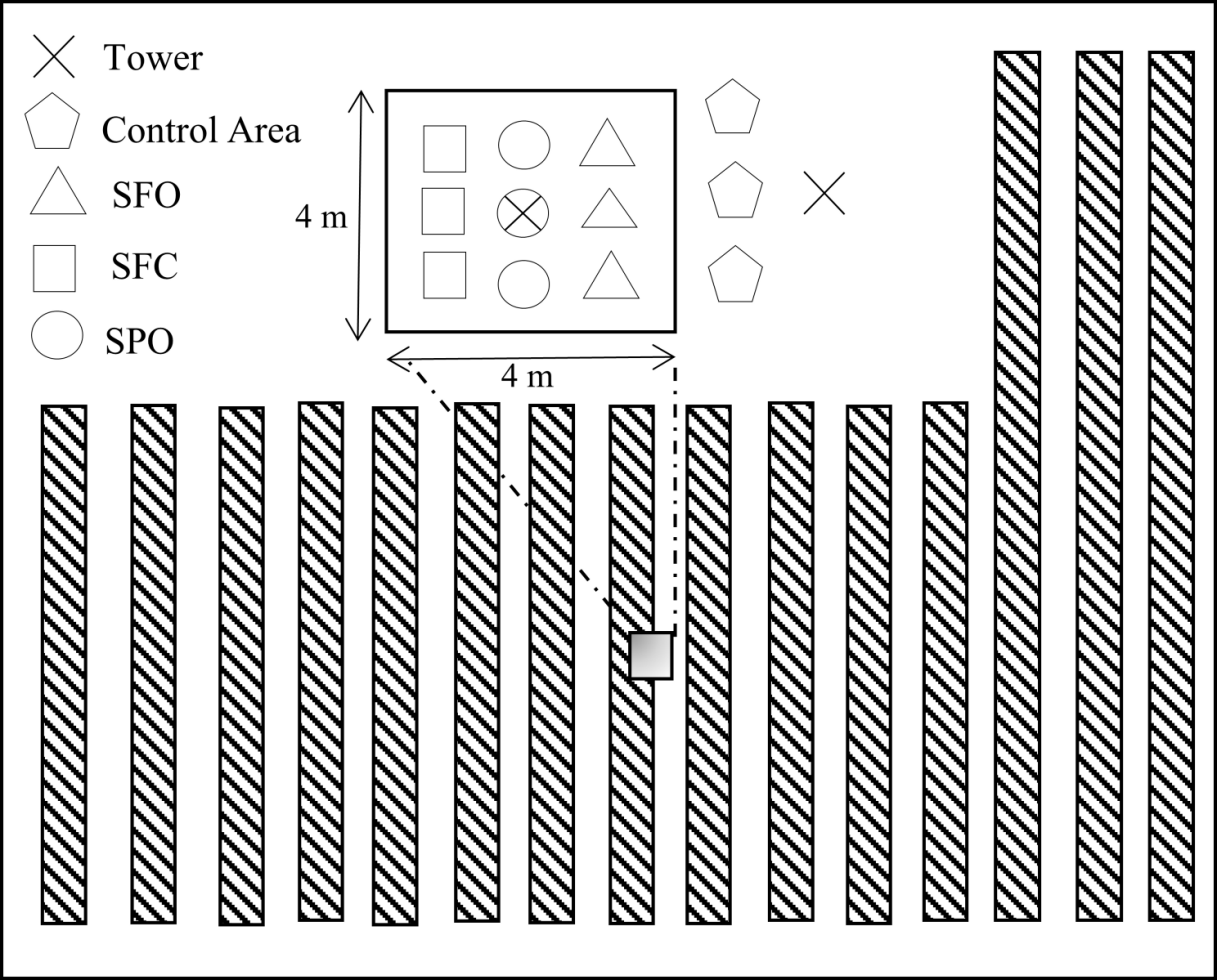
Plan view of experimental setup in solar array area showing locations of towers and neutron probe access tubes for: Solar Fully Covered (SFC), Solar partially open (SPO), Sky Fully Open (SFO), solar measurements are almost 70 meters apart from control area.

The field study was performed on a six acre agrivoltaic solar farm and sheep pasture near the Oregon State University Campus (Corvallis, Oregon, US.). The PhotoVoltaic Panels (PVPs) have been arranged in east–west orientated strips, 1.65 m wide and inclined southward with a tilt angle of 18^o^. PVPs have been held at 1.1 meters above ground (at lowest point) and the distance between panels is 6 meters as shown in Fig 1) e. The whole solar array system has a capacity of 1435 kilowatts (http://fa.oregonstate.edu/sustainability/ground-mounted-photovoltaic-arrays). As shown in Fig 1, the data were collected from localized zones (described hereafter) including areas below solar panels and a control area outside the agrivoltaic system. The pasture below the solar panels and the control areas were in the same paddock that was actively grazed by sheep. Exclusionary plots, to eliminate grazing pressure, were maintained with fencing. The total size of the fenced areas was limited by agricultural activities. The pasture was not irrigated, and typically experiences water stress mid-summer. The soil classification for >70% of the pasture area (control and agrivoltaic system) is Woodburn Silt clay [20]. The control and treatment plots were located within Woodburn Silt clay classification areas. The intent of the field measurements was to minimize uncontrollable differences between the treatments and control (e.g. solar forcing, soil types) and minimize impact on agricultural activities. Thus, the distance between the treatment site and the control site was kept minimum. The observations within the treatment site were further divided into three sub-treatments (Fig 2): (1) Sky Fully Open area between panels (SFO), (2) Solar Partially Open between panels (SPO) and (3) Solar Fully Covered area under panels (SFC). SFO areas are between the edges of installed PV panels and experienced full sun. Shadow length calculation also confirms no shade covers the SFO zone [21]. SPO areas are in the penumbra and experienced episodic shade. SFC areas are directly beneath the PV panels and experienced full shade. Data from these sub-treatments were compared to the data collected from the control area outside the agrivoltaic array, where each measurement was replicated.

Shadow length (L) is calculated [20]based on the sun latitude, solar panel height, day and time of the year the and it changes from 1.1 meters to 1.4 meters for May, June, July and August of 2015 which makes the SFO no shadow zone.Data were collected continuously in all areas from May-August 2015. Air temperature, relative humidity, wind speed and wind direction measurements were collected on 1 minute intervals. Soil moisture profiles were collected three times each week, and biomass samples were collected at the end of the observation period. Details associated with each set of measurements are explained in the following sub-sections.

### 2.1 Microclimatological measurements

Two atmospheric profiling stations were installed 70 meters apart: one in the control area and one near the center of the solar panel area. Micrometeorological variables were collected at four levels (0.5, 1.2, 2.0 and 2.7 m aboveground) in 1 minute intervals. The gathered variables were (1) air temperature (VP-3 Decagon Devices), (2) wind speed and directions (DS-2 Decagon Devices), (3) relative humidity (VP-3 Decagon Devices) and (4) net radiation (PYR Decagon Devices). Data were logged on EM50 data loggers (Decagon Devices). Temperature and humidity devices were calibrated in a chamber, and wind sensors were calibrated in a wind tunnel prior to installation. A Kolmogorov Smirnov test was used to detect differences in *distributions* of temperature, humidity, wind speed, wind direction, and down welling radiation between the solar array area and the control area. A two tailed t-test was used to detect differences in the *mean* temperature, humidity, wind speed, wind direction, and down welling radiation between the solar array area and the control area and standard deviation results was measured to quantify the amount of dispersion of a set of data values.

### 2.2 Soil moisture measurement

The soil moisture was obtained using a neutron probe device (503 DR hydro-probe Campbell Pacific Nuclear International Inc. BoartLongyear Corporation (CPN), Concord, California, USA). These data were gathered at six depths for each sampling location (0.1 m to 0.6 m in 0.1 m intervals). Fig 2 shows a plan view where nine neutron probe access tubes for soil moisture measurements were installed in the solar area. Three access tubes were installed in each sub-treatment: SFO, SPO, and SFC respectively. Three access tubes were also installed in the control area. Neutron Probe readings were taken approximately every three days. A standard count was taken prior to sampling each day to calibrate data readings. Three neutron counts were averaged for each individual measurement (a single depth in a single tube). This count was normalized by the standard count, and the normalized count was calibrated to soil moisture. Within each sub-treatment, data at the same depths are averaged to determine the soil moisture profile and error-bars. The result is a soil moisture profile with measurements at 0.1, 0.2, 0.3, 0.4, 0.5, and 0.6 m for each sub-treatment and the control every three days. Neutron probe readings at the 0.1m depth for all sub-treatments and the control were adjusted to account for possible neutron losses to the atmosphere [22]. Two-way ANOVA was used to test the independence of the soil moisture measurements with respect to zoning (the control, SFO, SPO, and SFC) and depth.

### 2.3 Biomass measurements

Above-ground biomass was collected on the 28^th^ of August. Six 1m by 1m quadrants were collected from within the fenced areas for each sub-treatment and the control. Harvested biomass was dried for 48 hours in a 105 ^o^C oven and weighed. The Daubenmire method [11] was used to study grass species diversity at the end of July. Six transects in the control and one transect in the solar array were performed. For each transect, a random number was drawn (from 1–10) to determine the final location of each 1m x1m quadrant. Six quadrants were collected in each transect resulting in a total of 42 samples. In each quadrant, the coverage, by species, was determined visually.

## 3 Results and discussions

### 3.1 Micrometeorological variables

Using a Kolmogorov Smirnov test, a two tailed t-test, standard deviation and William Watson test[23] for wind direction showed subtle but statistically significant differences. Significant differences in mean temperature were found in readings taken closest to the PV panel surfaces at the 1.2 m and 2.0 m elevations. No significant differences were observed at the lowest (0.5 m) or highest (2.7 m) elevations. Note that the magnitude of these mean temperature differences are smaller than those reported from simulation studies [5–9]. Significant differences in mean relative humidity and wind speed were found for all measurement heights. Solar radiation below the solar panel installation height was significantly reduced (as expected) and the incoming solar radiation measured at a height above the solar panels was found to be statistically significant (unexpected) but the difference relatively small. The distribution of wind direction was significantly altered at all heights, and the mean wind speed was significantly different at all heights. A summary of the p-values from all statistical tests is shown in Table 1. Standard deviation values were big due to diurnal changes of micro climate variables during the day.

**Table 1:** Mean/Std and p-values from solar panel and control area Two-sample Kolmogorov-Smirnov, t tests and William Watson test.

Elevation (m)		0.5	1.2	2.0	2.7
**Temperature** **(°C)**	Mean/Std (solar panel area)	18.34/7.87	18.03/8.06	18.30/7.39	18.37/7.65
	Mean/Std (control area)	18.27/7.85	18.32/8.31	18.36/7.47	18.11/7.64
	p-values (KS test)	0.9964	0.9964	1.0000	1.0000
	p-values (t test)	0.1527	**0.0000**	**0.0000**	0.5996
**Relative humidity** **(%)**	Mean/Std (solar panel area)	65.62/0.226	64.17/0.252	64.29/0.209	64.92/0.230
	Mean/Std (control area)	66.23/0.234	66.38/0.242	64.89/0.222	65.37/0.223
	p-values (KS test)	**0.0004**	0.3611	0.7014	0.6703
	p-values (t test)	**0.0000**	**0.0000**	**0.0000**	**0.0118**
**Wind speed** **(m/s)**	Mean/Std (solar panel area)	0.5471/0.506	0.4880/0.427	1.3777/1.083	1.0889/0.909
	Mean/Std (control area)	0.8752/0.665	0.6384/0.520	1.1313/0.859	0.9726/0.757
	p-values (KS test)	0.9579	1.0000	0.8497	0.9964
	p-values (t test)	**0.0000**	**0.0000**	**0.0000**	**0.0000**
**Solar radiation (W/m2)**	Mean/Std (solar panel area)	-	59.53/96.65	-	275.72/322.59
	Mean/Std (control area)	-	328.26/407.93	-	271.58/323.34
	p-values (KS test)	-	**0.0099**	-	0.9597
	p-values (t test)	-	**0.0000**	**-**	**0.0054**
**Wind direction** (°)	Mean/Std (solar panel area)	196.29/107.71	220.96/102.32	211.83/101.68	206.11/96.65
	Mean/Std (control area)	210.54/102.29	196.82/121.16	211.87/95.91	182.13/115.97
	p-values (WW test)	**0.0000**	**0.0000**	**0.0000**	**0.0000**

**Fig 3:**
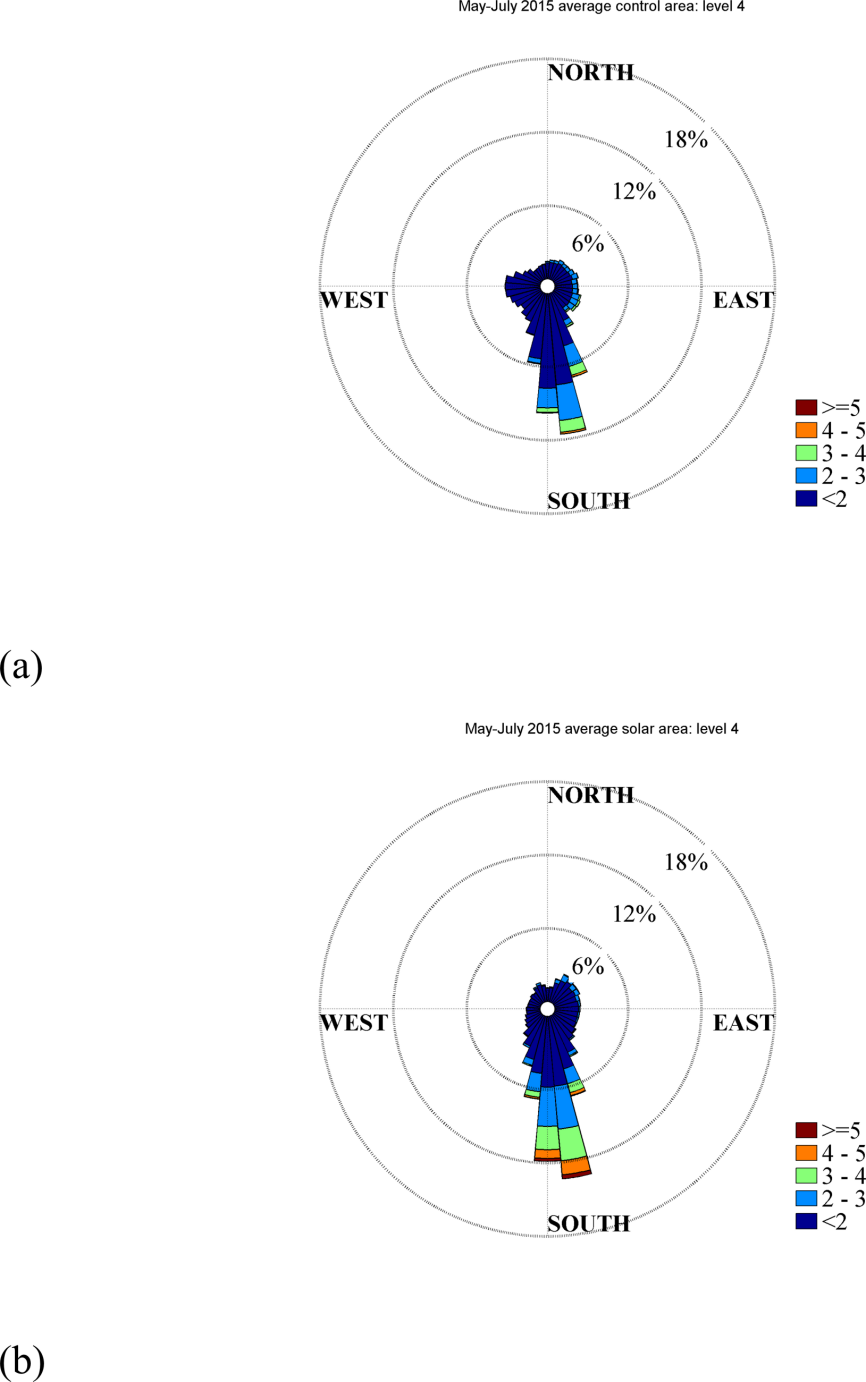
Wind rose plots for control (upper) and solar areas (lower) for May-August average wind directions. The data are for elevation 2.7 m.

Wind direction data at 2.7 m above ground level is shown in Fig 3 to illustrate the alterations in the wind direction. For the sake of brevity, only one height is presented in this manuscript, but all heights are shown in Supporting Information (Figure A in S1 Appendix). Fig 3 shows a histogram of incident wind direction plotted as a function of direction. Longer spokes indicate that that particular direction is more probable. Each spoke is divided and colored according to the strength of the wind (wind speed). For example, a long blue spoke would indicate that light winds from that direction are common. We can conclude from Fig 3 that the wind direction in the control area is distributed among many incident angles, while the wind direction within the treatment is oriented predominantly from the south. That is, the wind direction within the treatment area reorients with solar panels such that the wind is from south to north. The panels do not act as ‘canyons’ and orient the wind along their rows (a common occurrence in urban flows for example)[24]. Rather, the wind is reoriented perpendicular to the solar array’s rows. The authors believe that the local increase in temperature near the solar panel surface results in a buoyant force that causes local anabatic flow up the panel surfaces. Each anabatic flow on each PV row has a vector component perpendicular to the solar panel row orientation, and the entire solar farm acts like a ‘Fresnel slope’ that reorients the flow. The total buoyant force is enough to accelerate the flow directionally, and contributes the increase in wind speed above the panels. Flow acceleration around a bluff body (PV panel) also contributes to increased wind speed above the solar panels. Increased drag due to the ‘solar canopy’ is likely the cause of the reduced speed below the solar panels. Note that the most common wind speeds are weak (<2m/s), and it is unclear if this wind direction shift would be a robust result for windy locations. Higher wind speeds are also observed to reorient in Fig 3; however, the number of occurrences are limited.

### 3.2 Soil moisture data comparisons

The horizontal axis shows the Day of Year (DoY) of the data collection in 2015 and vertical axis is the volumetric soil moisture in vol/vol. Independence was determined with a p-value of less than 0.01 for all depths and zones by two-way ANOVA test. The soil moisture is near saturation for all depths and all treatments at the start of observation. That is, all areas had nearly identical initial soil moistures. The differing rates of soil water depletion in the three sub-treatments and the control led to dramatic differences in late season soil moisture.

The soil moisture in the SFO area is depleted more rapidly than the SPO, SFC or control areas. This result is intriguing since the SFO area and the control experience similar incident solar radiation. Thus, the SFO must have a different energetic balance despite similar exposure to direct solar energy. We hypothesize that this difference in rate of soil moisture loss is a result of the longwave radiation transfer. The SFO will experience incident long wave radiation from the adjacent PV panels. These PV panels would also reduce the sky view factor of the SFO area. In contrast, the sky view in the control area is unobstructed. Thus, we infer that the total net long wave and net shortwave radiation both play an important role in the energetics and evaporation in the SFO area. The complete long and short wave radiation budgets within an agrivoltaic system will be explored in future study.

**Fig 4:**
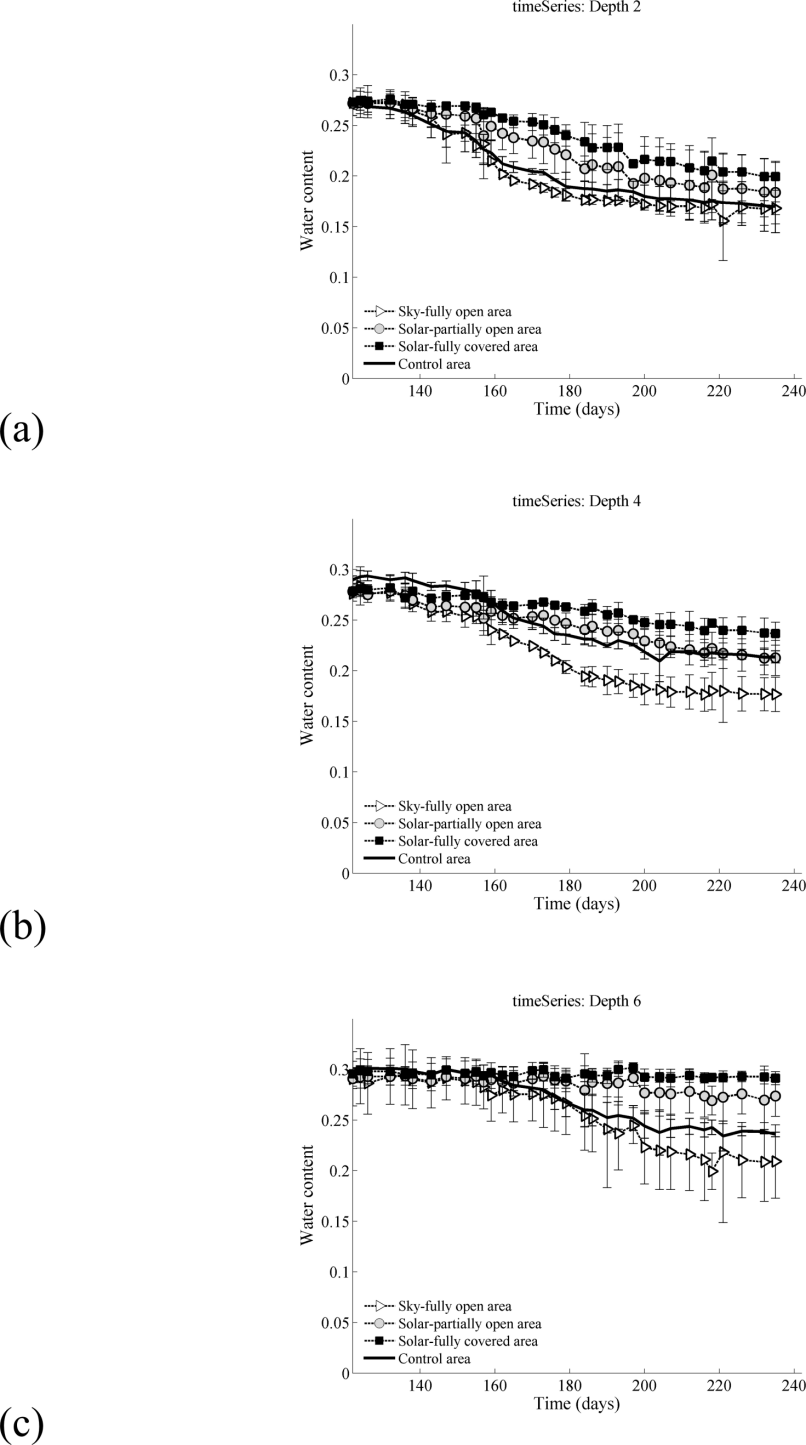
Soil moisture time series (a) 0.2m, (b) 0.4m and (c) 0.6m. For more information: there was 40 mm precipitation over the observation period, i.e. May-Aug 2015.

Time series of the soil moisture at 0.2 m, 0.4 m and 0.6 m are presented in Fig 4 in subpanels a-c. Time series of soil moisture at 0.1 m, 0.3 m and 0.5 m can be found in Supporting Information (Figure A in S2 Appendix). Soil moisture is most persistent in the SFC area and remains available for a larger portion of the growing season. The soil moisture at 0.6 m depth remained close to saturation (0.3 vol/vol) for the entire season which implies that water is available at the bottom of the root zone over the period of observation Fig 4C. Overall the SFC area remained wetter than all other areas, including the control. This water availability is in stark contrast to the SFO area which was near saturation at the start of observation (~0.3 vol/vol) and depleted to ~0.2 vol/vol at the end of the season. This stark contrast in the moisture availability between the SFO and SFC creates an undesirable variability across the field and hints that shade uniformity may be an important consideration for the design of future agrivoltaic systems. The SPO area dries at a rate slower than the SFO but faster than the SFC and the control.

In other words, for most times and soil depths, the SFC had that highest soil moisture followed by the SPO, control and SFO respectively. It should be noted that the mean soil moisture across the SPO, SFO and SFC regions is similar to the control. But, the solar panels increase the local heterogeneity of soil water conditions, which results in some areas (SFC) having more persistent stores of soil water throughout the growing season.

**Fig 5:**
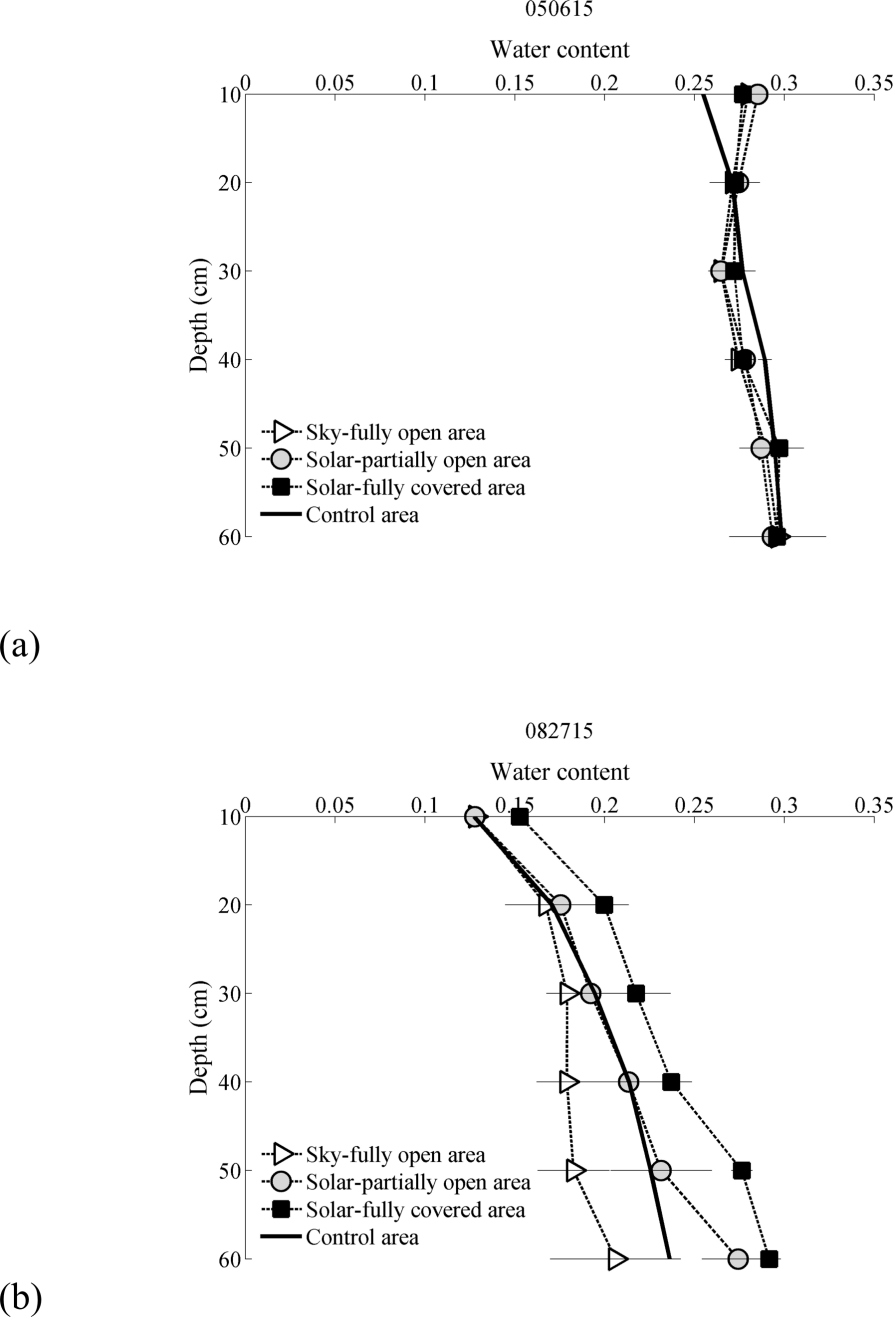
Selected normalized soil moisture profiles from data sampling to show the change in soil moisture through growing season, (a) May 06–2015 and (b) August 27–2015.

The soil profiles at the beginning and end of the observation period are shown in Fig 5 All areas were near saturation for all depths initially. By the end of the observation period, the soil moisture in the SFC zone was nearly twice the SFO. These measurements are separated by less than two meters spatially. All measured soil moisture profiles are available in Supporting Information (Figure A in S3 Appendix).

### 3.3 Vegetation

Eight grass types were identified in the control pasture and five were identified in the solar farm area. A summary of the results is presented in Table 2. The most common species in the solar panel area was Alopecurus, a long-lived perennial that thrives in moist conditions. Alopecurus provides a “succulent and palatable forage” [25]. The most prevalent grass type in control area is Hordeum that has spikelet clusters that can enter nostrils and ear canals in mammals. Three types of grasses Calamagrostis, Cirsium and Dactylis were observed only in the control area. These grasses are only favored by sheep and cattle in the early stage of the grass before spine develops [26]. The causal factor for the diversity change between control and treatment requires further investigation.

**Table 2:** The results of biomass monitoring for different grass types in solar and control area.

Grass scientific name (common name)	Solar area (%)	Control area (%)
Hordeum (Foxtail barely)	10	25
Agrostis (Redtop bentgrass)	30	20
Alopecurus (Meadow foxtail)	50	7
Schedonorus (Tall rye grass)	5	9
Bromus (Foxtailbrome)	5	22
Calamagrostis (Reed grass)	0	6
Cirsium (Thistle)	0	10.5
Dactylis (Orchard grass)	0	0.5

**Fig 6:**
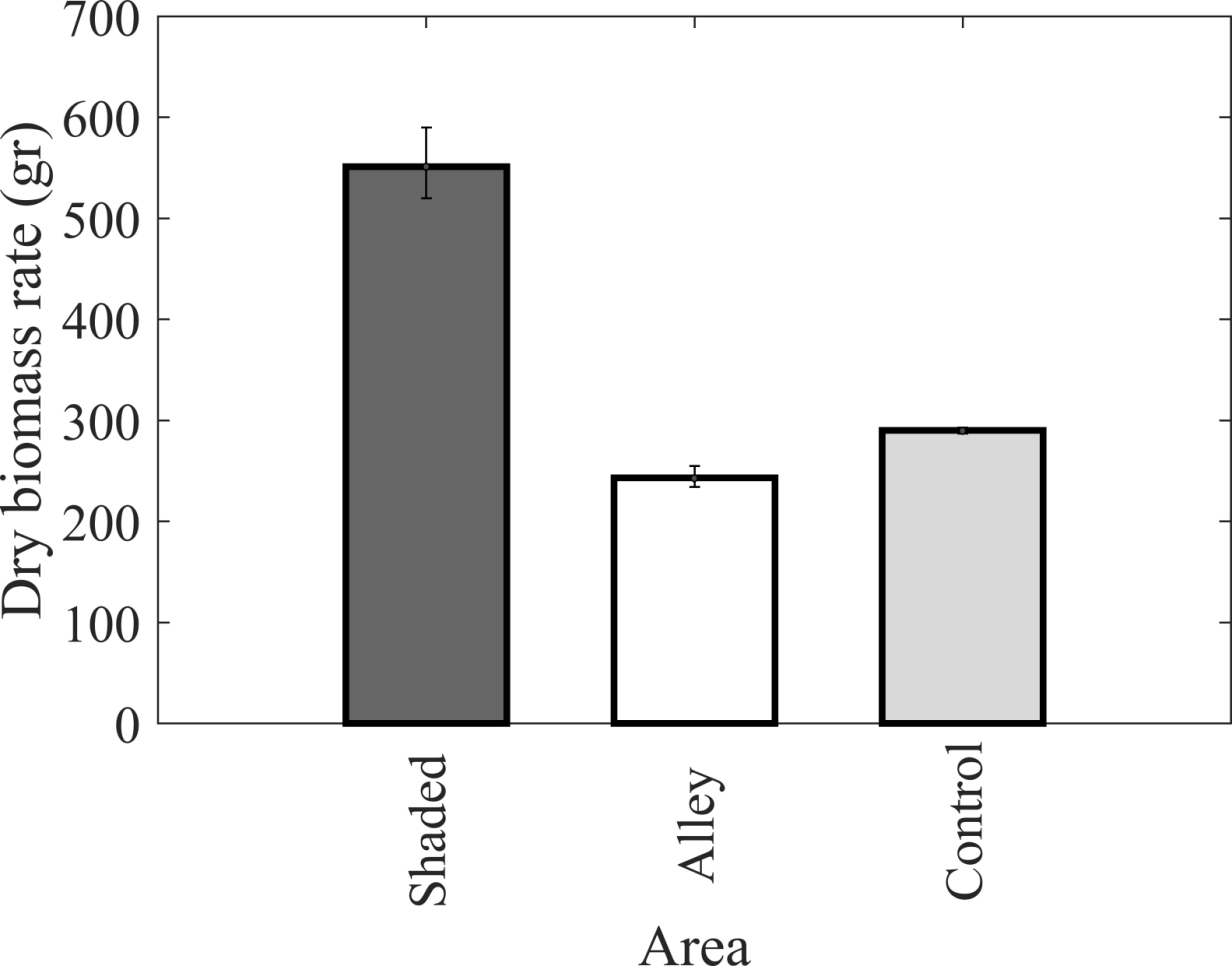
Dry biomass comparison in three places Solar Fully Covered (SFC), Sky Fully Open (SFO) and control area.

The harvested dry biomass at the end of the observation period is shown in Fig 6 Results show 126% more dry biomass in the SFC zone relative to the SFO zone and 90% more dry biomass in the SFC zone relative to the control. Although the sample size is small, difference between the SFC and the control were found to be significant, (p-value = 0.007). In addition, the difference between the SFC and the SFO were found to be significant, (p-value = 0.007).

### 3.4 Water usage

**Fig 7:**
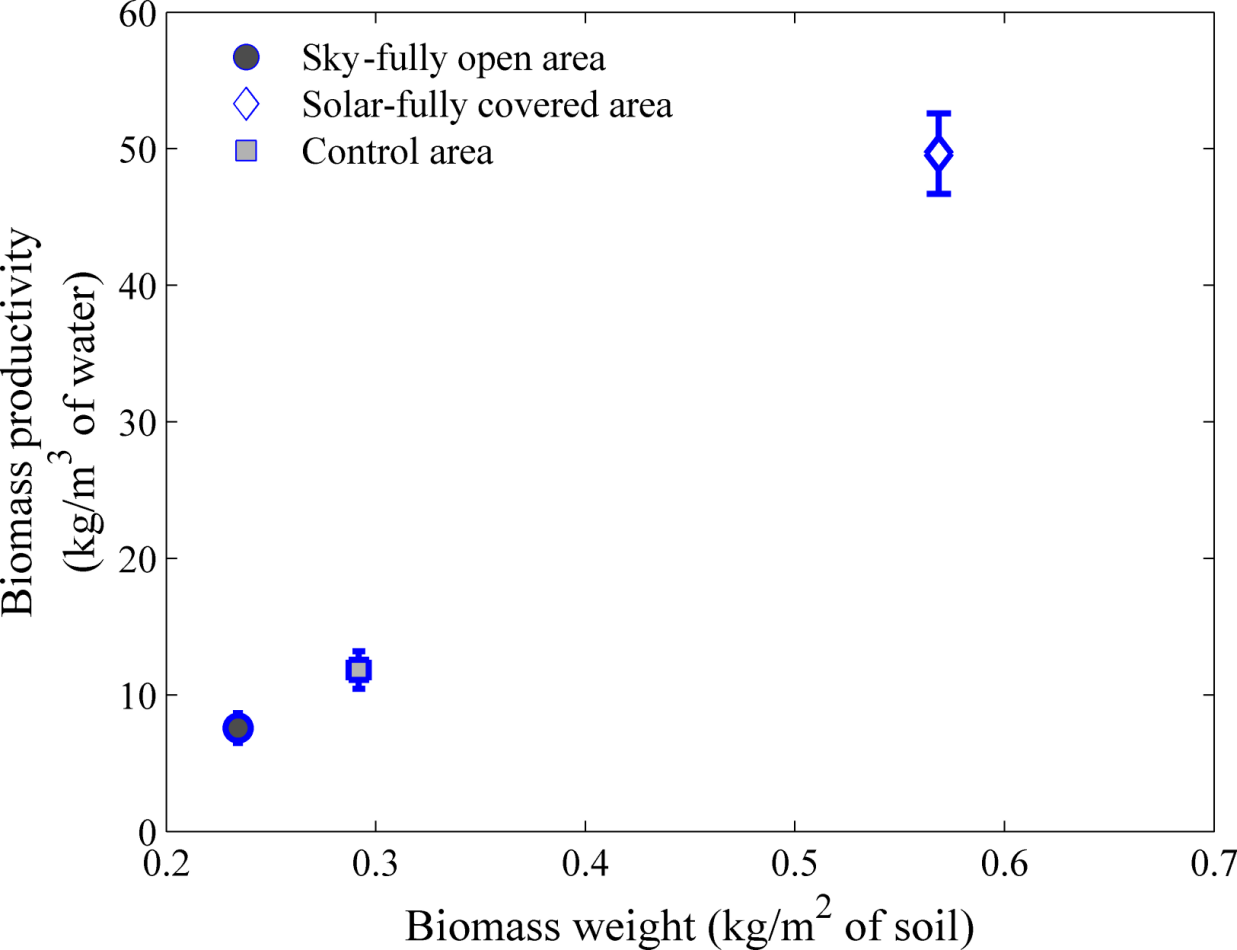
Biomass productivity in kg/ m^3^ of water.

Water usage was calculated based on difference in depth averaged soil moisture between the beginning (Fig 5(A) and end (Fig 5(B)) of the observation period. Averages are calculated by integrating soil moisture over soil depth from 10cm to 60cm. Water Use Efficiency (WUE) is then calculated as the biomass produced per unit of water used. Water use efficiencies in kg biomass/m^3^ of water against the biomass weight in control and SFO and SFC treatments are presented in Fig 7 ($\frac{WUE\;SFC–WUE\;Control\;area}{WUE\;Control\;area}$). The higher producing SFC treatment was also significantly more water efficient (328%).

The seasonal climate pattern at the site produces an initially saturated pasture and a a dry growing season. Initial water stores are depleted, through evapotranspiration (ET), and water scarcity occurs in the control and SFO areas. The shaded treatments (SFC and SPO) experience lower potential evapotranspiration (PET) due to decreased solar radiation throughout the observation period which resulted in a slower dry-down of the stored soil water. The decreased rate of dry-down in the SFC and SPO areas left soil water stores available throughout the observation period and allowed pasture grasses in the SFC and SPO to accumulate a significantly greater biomass. The reduced PET in the SFC and SPO treatments also contributed to an increase in water use efficiency of the pasture grasses. That is, a ‘water limited’ area, in a Budyiko [27] sense, could be considered as an area of ‘solar excess.’ By harvesting this solar excess with solar panels, PET is reduced. Taken to an extreme it is possible to shift the aridity such that the shaded area becomes energy limited. Thus there must exist a shading level, for a water limited area, where PET and AET would be in balance. We would not expect a similar outcome in ‘energy limited’ areas (Budyko sense) as observed by Armstrong et al. [8]. In this case, there is no solar excess and the PET is already equal to the AET. If solar arrays were placed above growing plants in ‘energy limited’ conditions we would expect that the total biomass production would decrease, consistent with the findings of Armstrong et al. [16].

## 4 Conclusion

Typical agricultural operations manage multiple on-farm resources including soil, nutrients and water. This study suggests that the on-farm solar resource management could also be implemented for productive benefits. Water limited areas are most likely to benefit as solar management reduces PET and consequently the water demand. Not all crops will be amenable to solar management, and the economics of active solar management with PV panels needs further study. But, semi-arid pastures with wet winters may be ideal candidates for agrivoltaic systems as supported by the dramatic gains in productivity (90%) observed over the May-Aug 2015 observation period at the Rabbit Hills agrivoltaic solar array. These net benefits were largely achieved through an increased water use efficiency in the shaded areas of the field which left water stored in the soil column available throughout the entire observation period. Extreme heterogeneity and spatial gradients in biomass production and soil moisture were observed as a result of the heterogeneous shade pattern of the PV array. Future agrivoltaic designs should eliminate this heterogeneity by optimizing PV panel placement to create a spatially uniform shadow pattern. A spatially uniform shadow pattern would foster uniform biomass accumulation benefits. The agricultural benefits of energy and pasture co-location could reduce land competition and conflict between renewable energy and agricultural production. Reduced or eliminated land completion would open new areas for PV installation. Local climatic effects of agrivoltaic installations were statistically significant but subtle, however the regional climatic impacts (e.g. rainfall patterns) of large scale agrivoltaic instillations are still unclear and should be the subject of further study.

## Supporting information

S1 Appendix Figure A: Wind rose plots for four level heights.
(DOCX)

S2 Appendix Figure A: Soil moisture time series (a) 0.1m, (b) 0.3m and (c) 0.5m. For more information: there was 40 mm precipitation over the observation period, i.e. May-Aug 2015.
(DOCX)

S3 Appendix Figure A: Selected normalized soil moisture profiles from data sampling to show the change in soil moisture through growing season: May 06–2015 to August 27–2015. The dates are mentioned on top of each figure with mmddyy format.
(DOCX)
