# Supplementary figures and images for: Remarkable agrivoltaic influence on soil moisture, micrometeorology and water-use efficiency
Source: PLoS One. 2018 Nov 1;13(11):e0203256. doi: 10.1371/journal.pone.0203256 (PMC6211631; doi:10.1371/journal.pone.0203256)

# Appendix S1

| 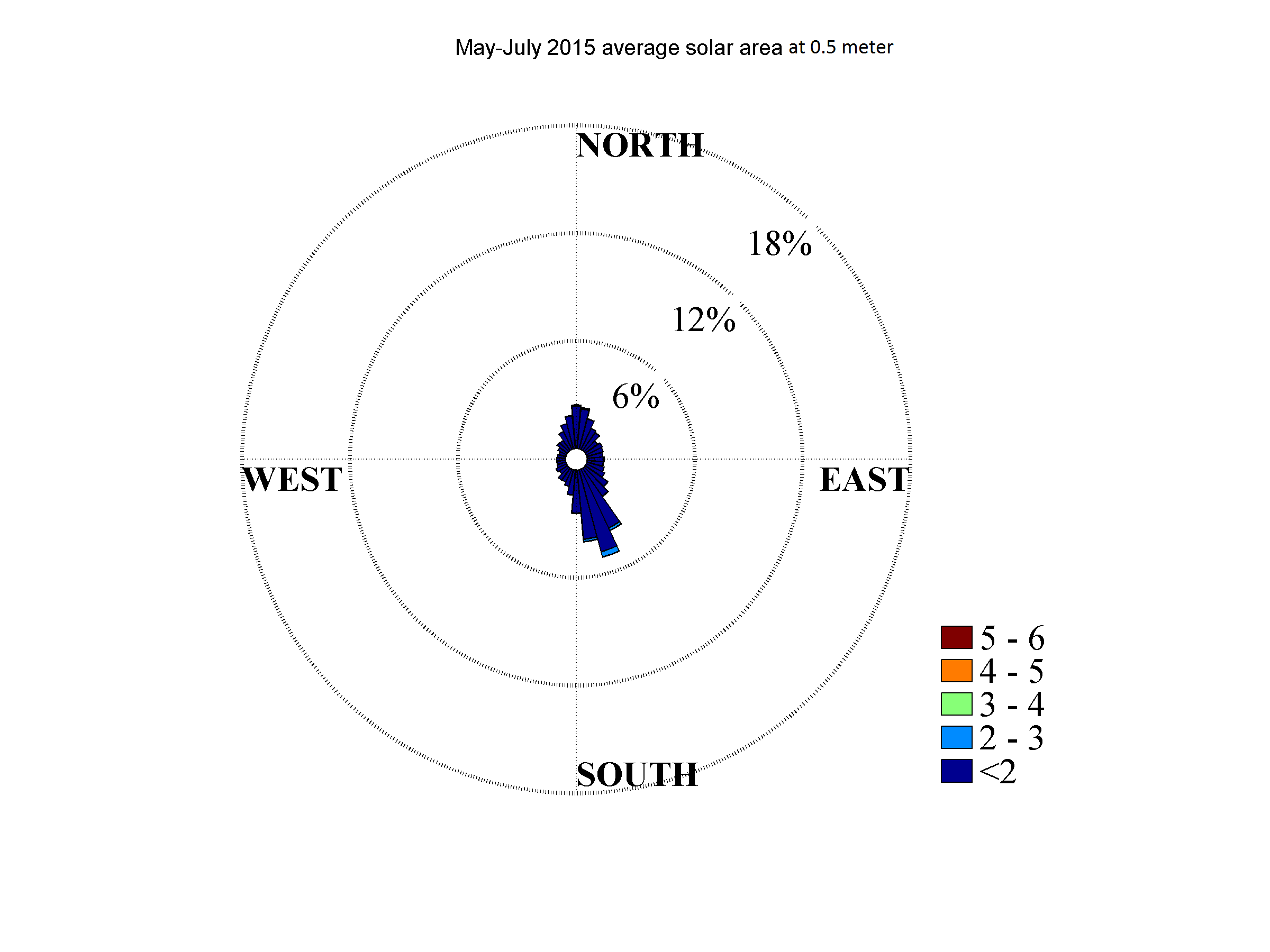 | 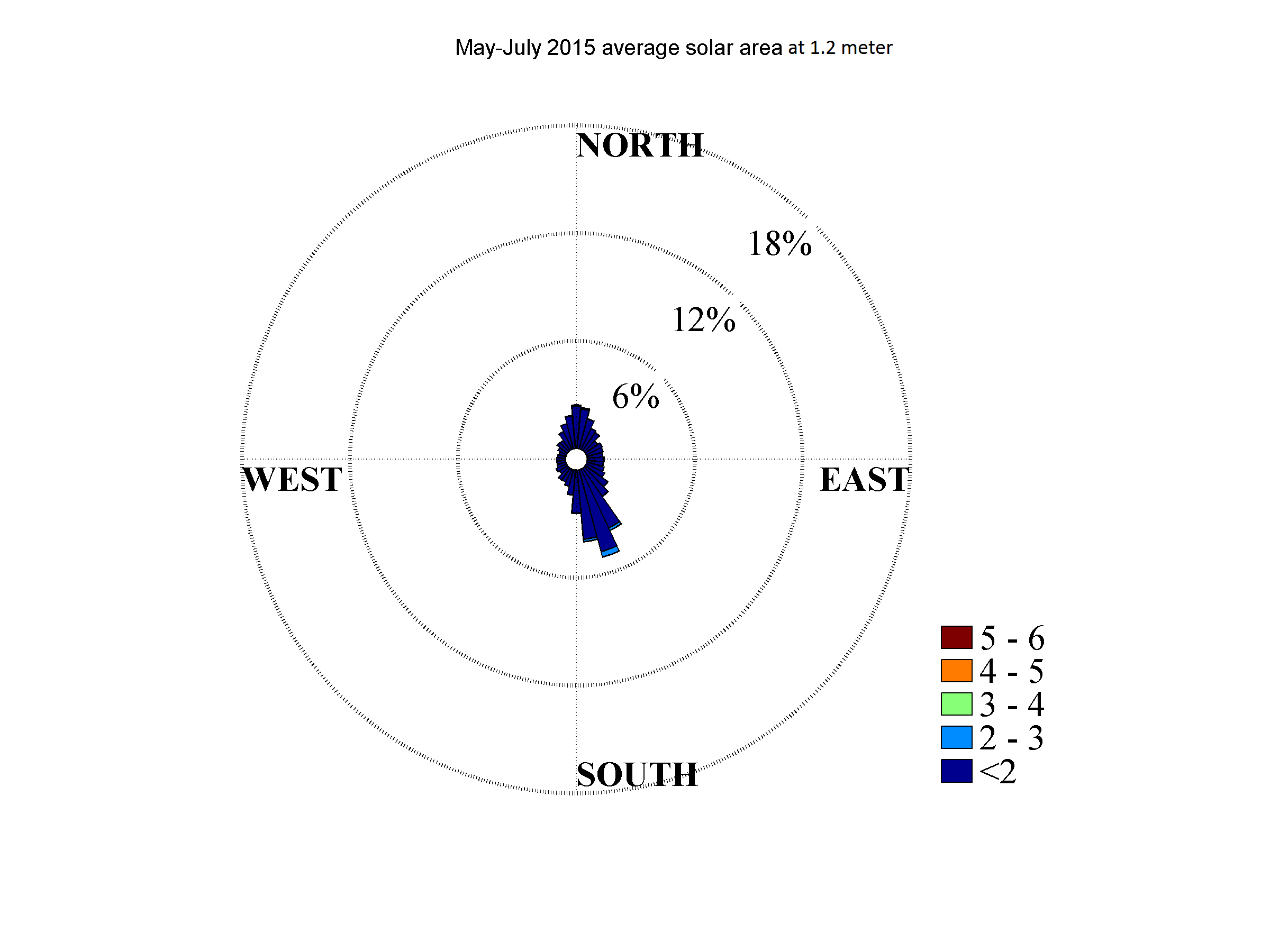 |
| --- | --- |
| 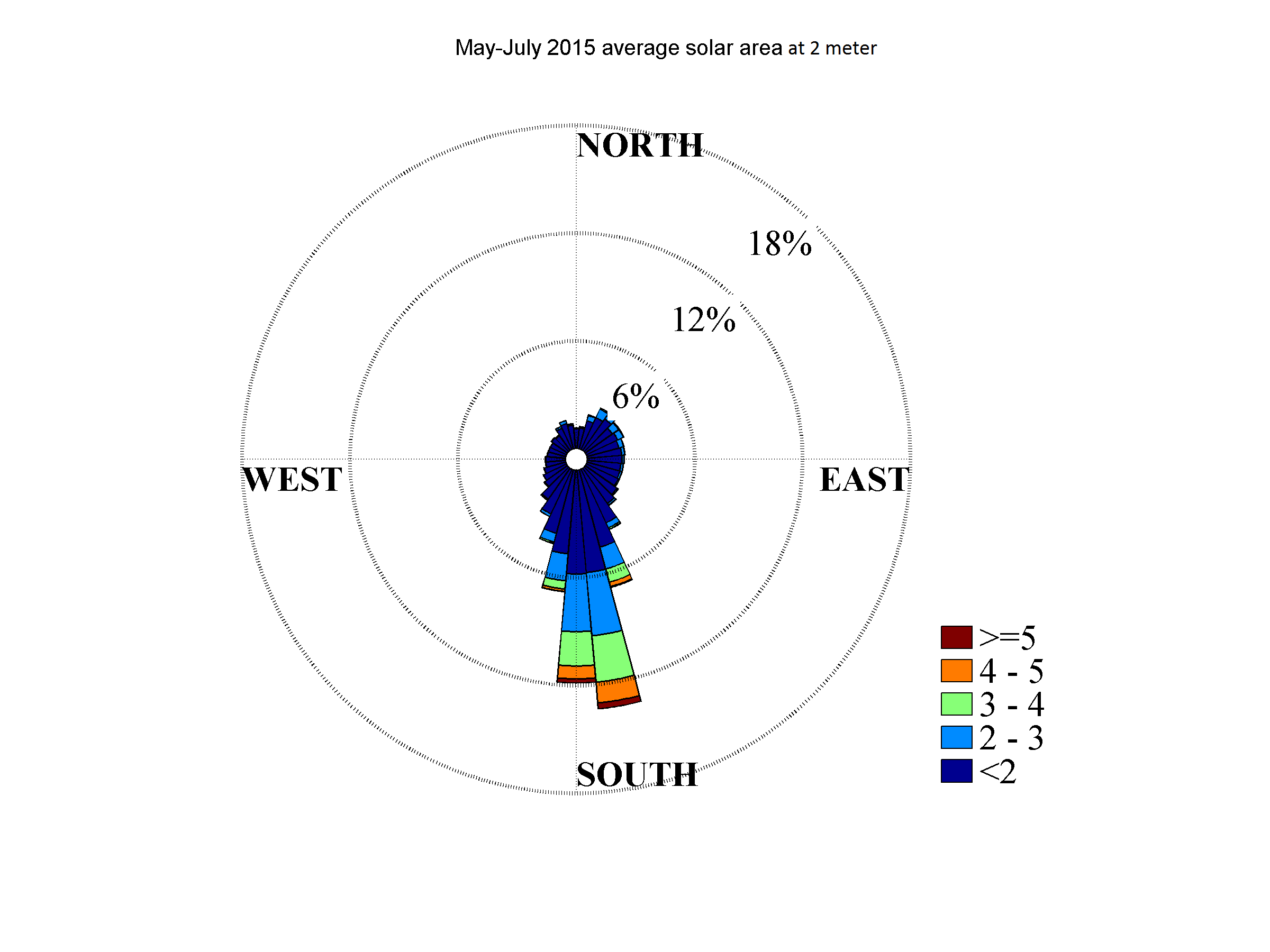 | 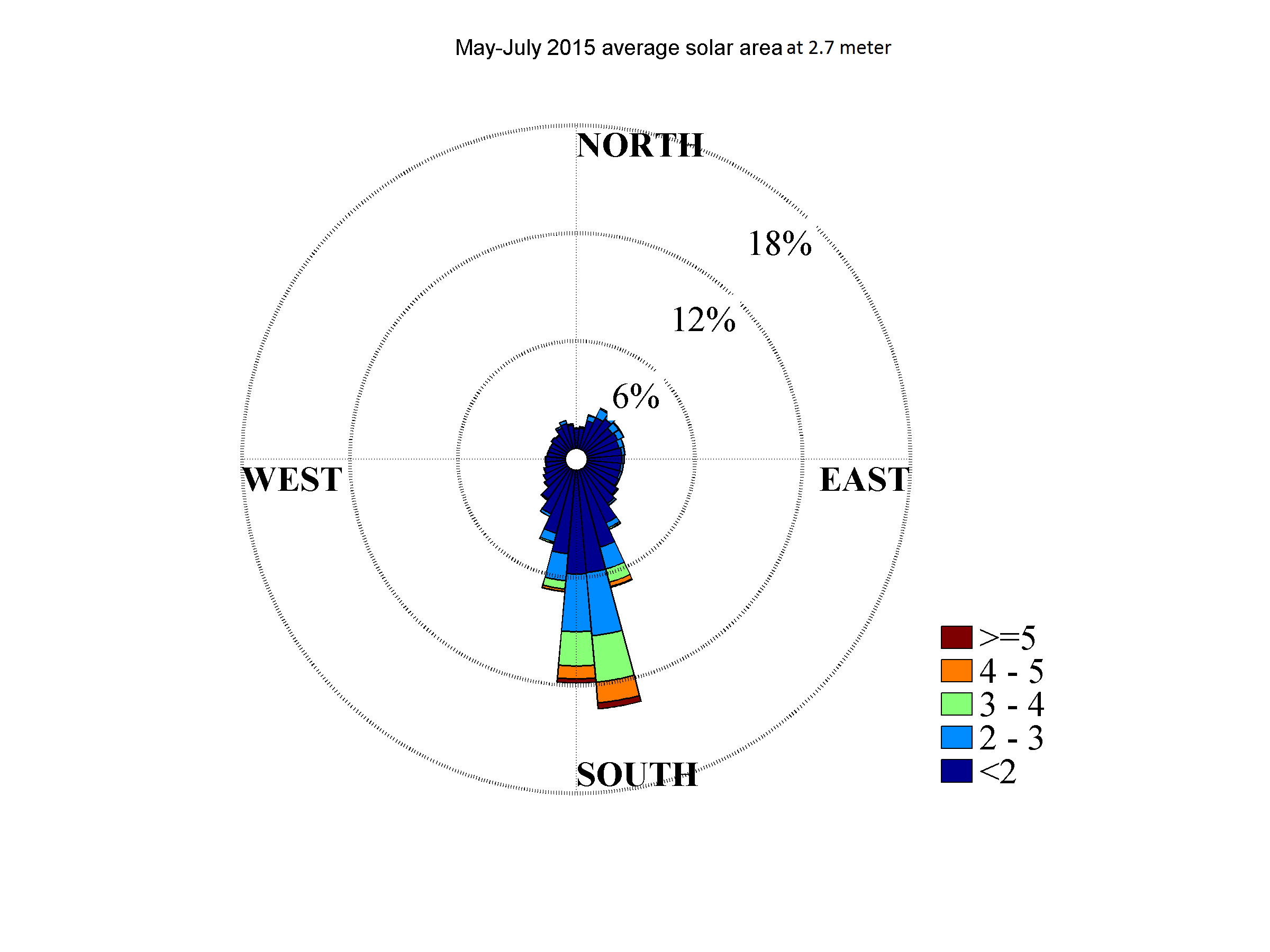 |

Supplement: S1 Appendix — Figure A: Wind rose plots for four level heights. (DOCX) [file pone.0203256.s001.docx]

# Appendix S2

| 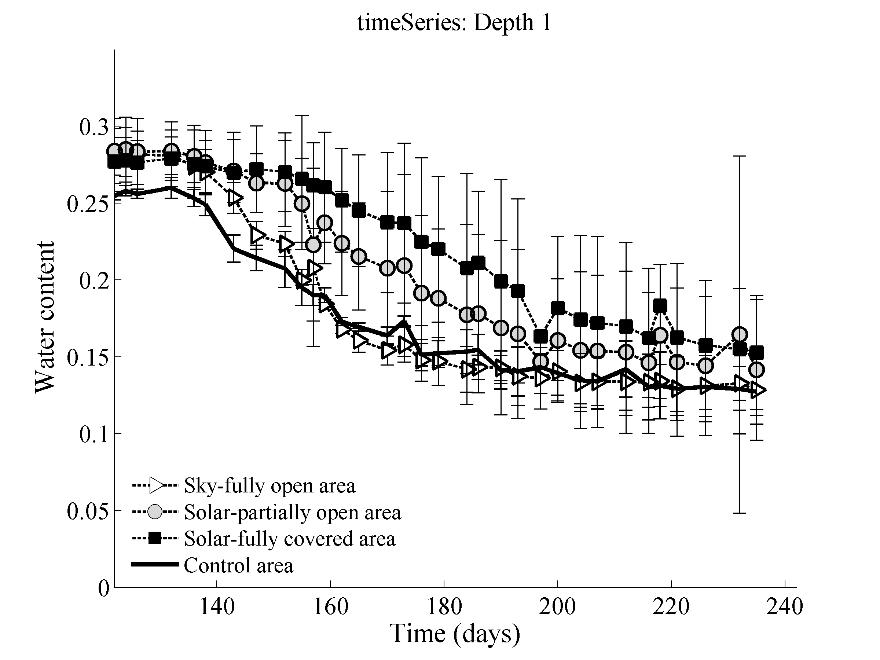   1. 0.1 m |
| --- |
| 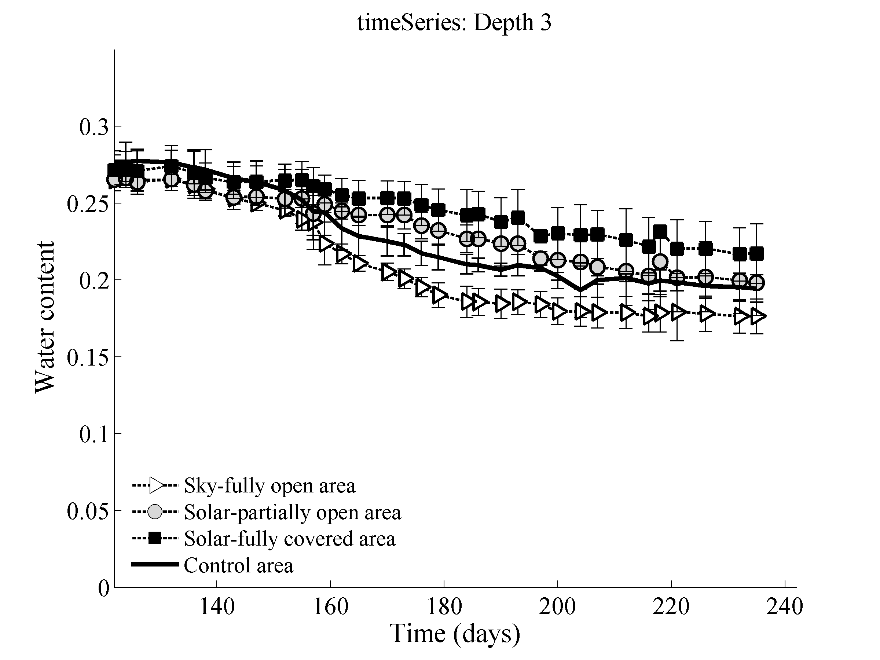   1. 0.3 m |
| 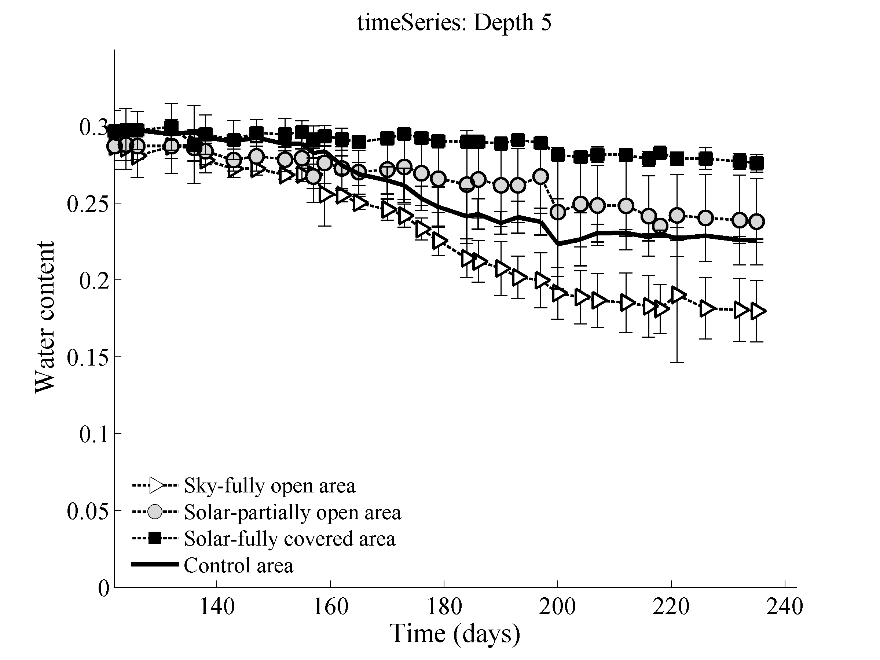   1. 0.5 m |

Supplement: S2 Appendix — Figure A: Soil moisture time series (a) 0.1m, (b) 0.3m and (c) 0.5m. For more information: there was 40 mm precipitation over the observation period, i.e. May-Aug 2015. (DOCX) [file pone.0203256.s002.docx]

# Appendix S3

| 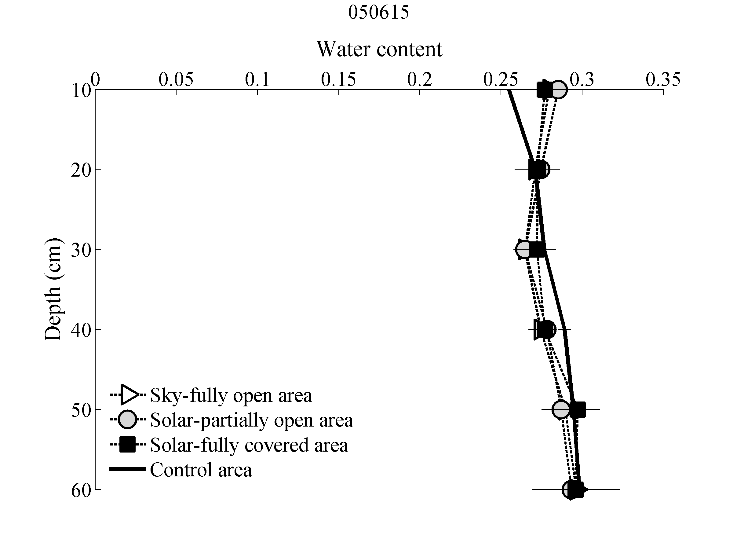 | 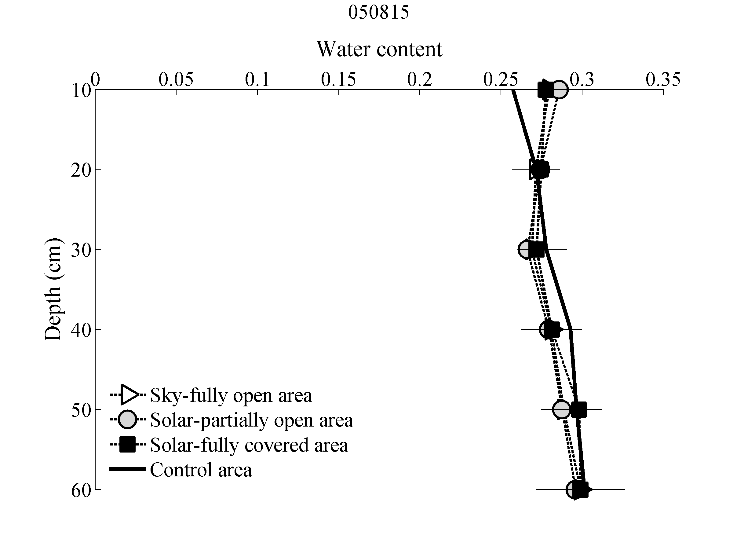 |
| --- | --- |
| 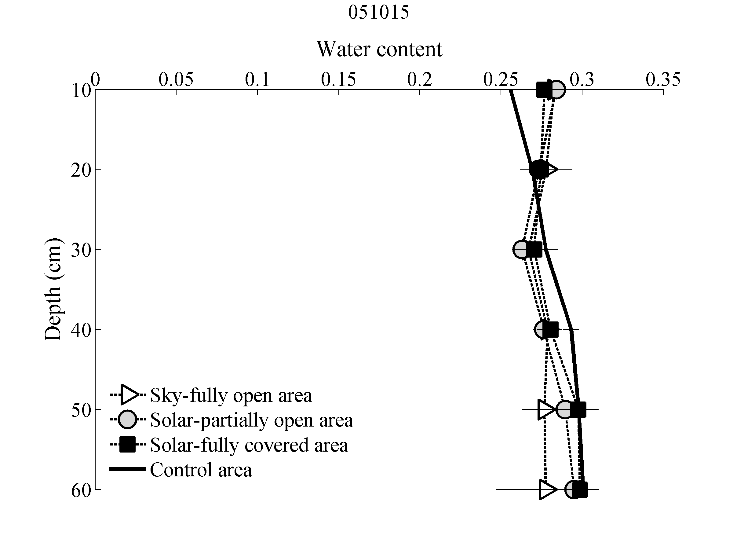 | 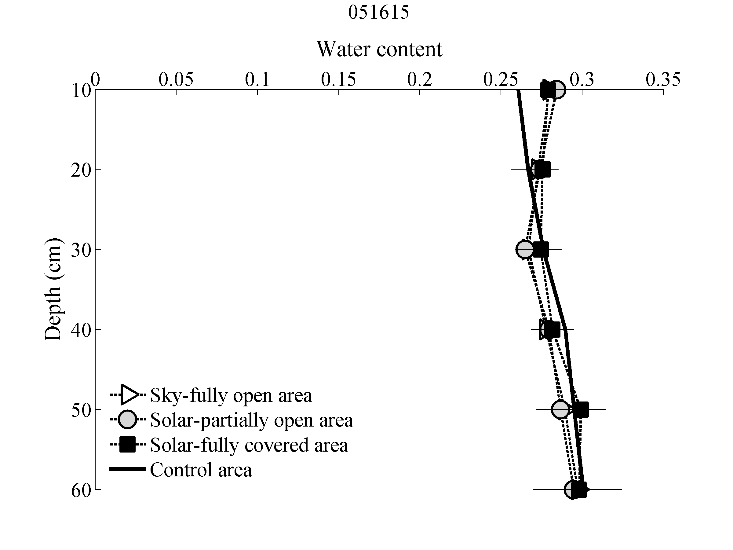 |
| 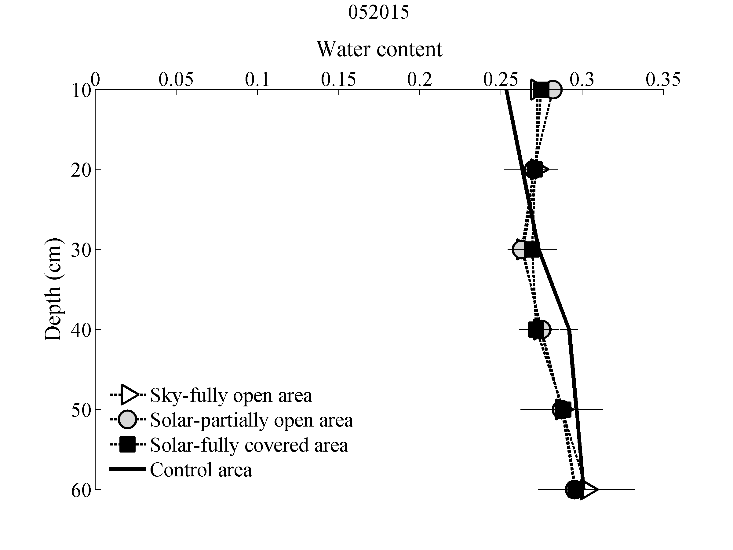 | 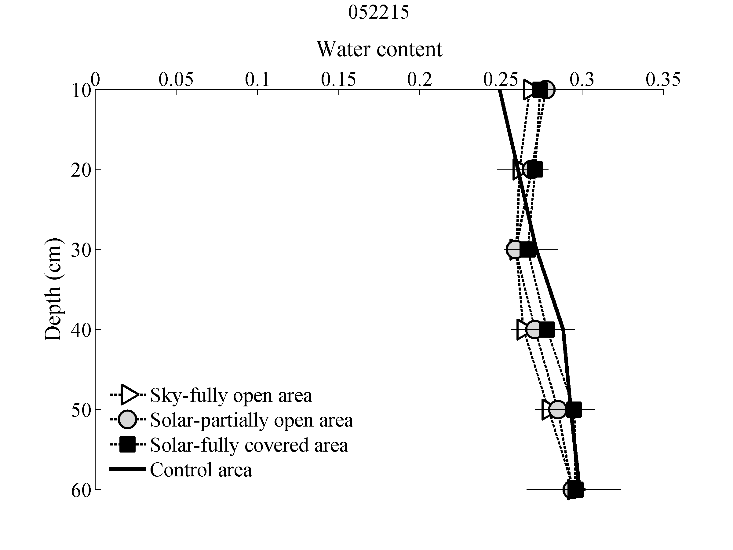 |
| 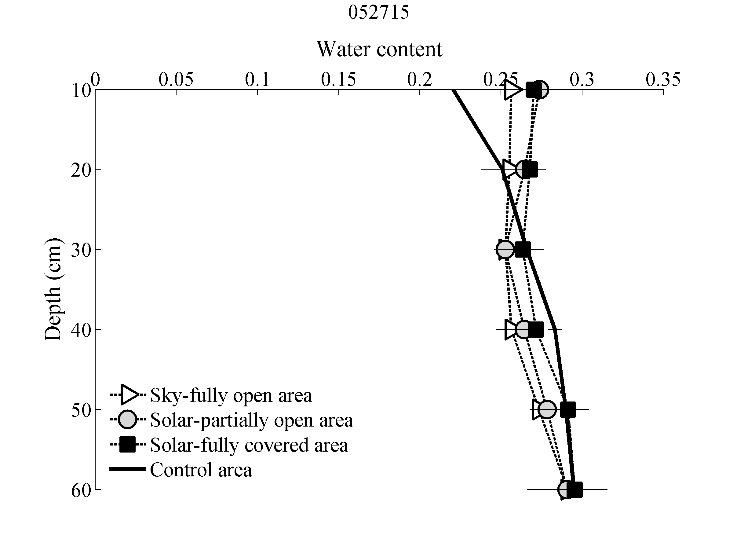 | 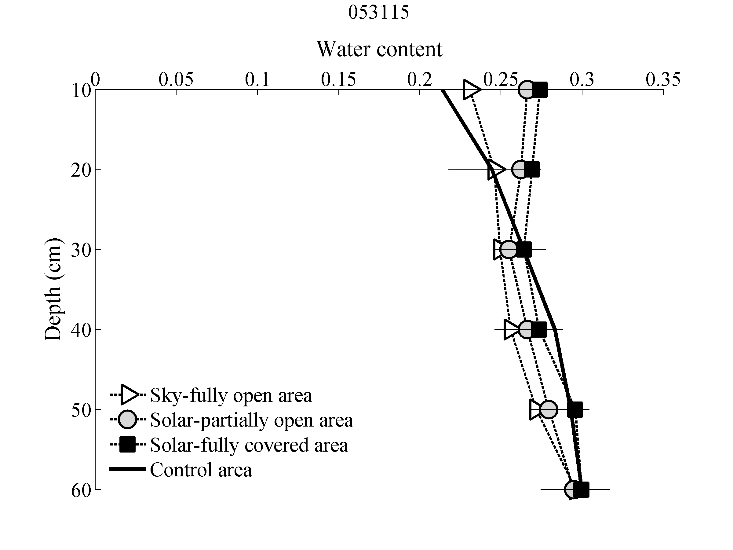 |
| 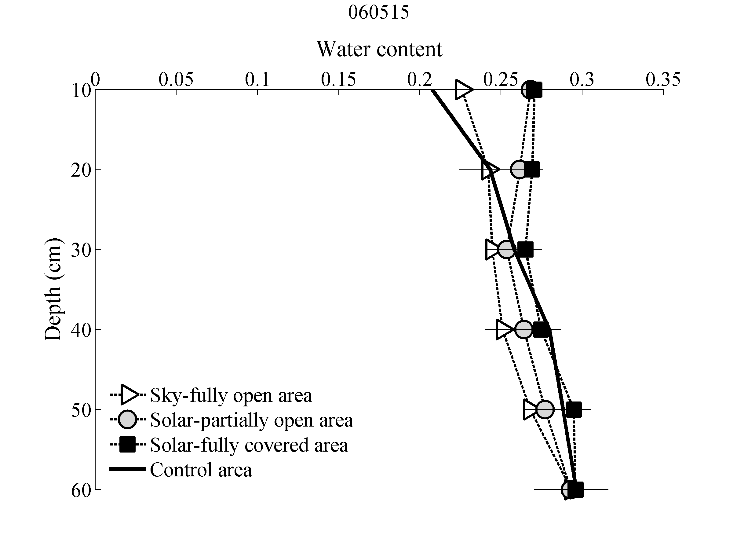 | 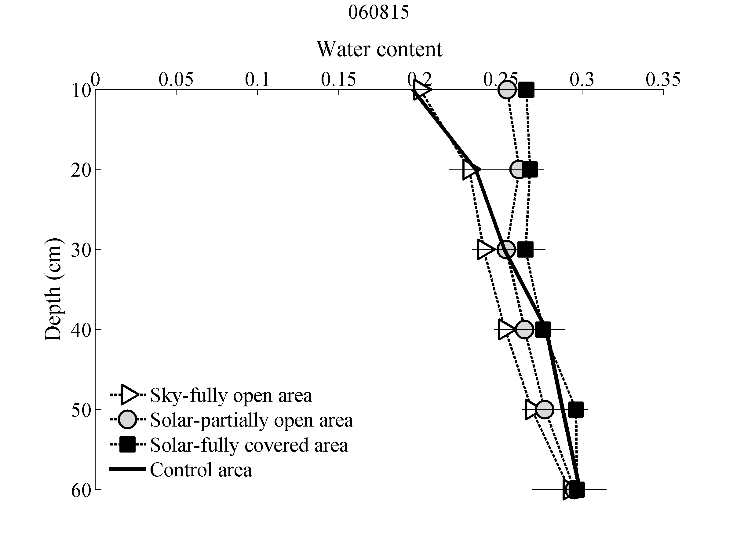 |
| 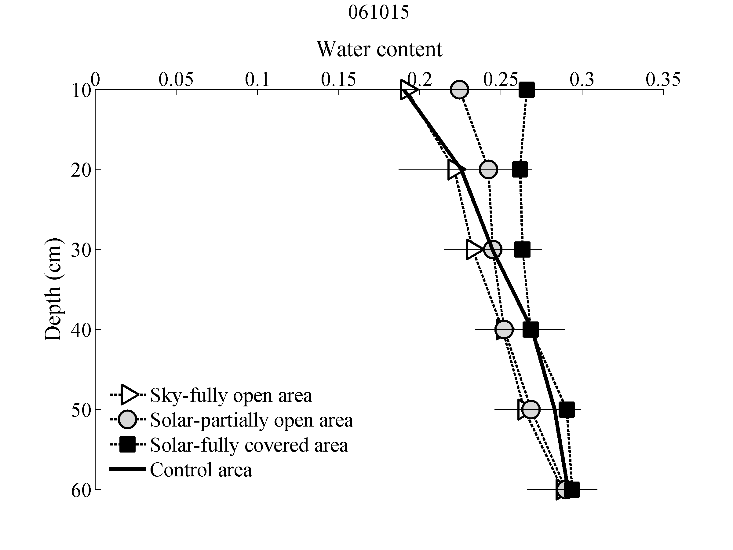 | 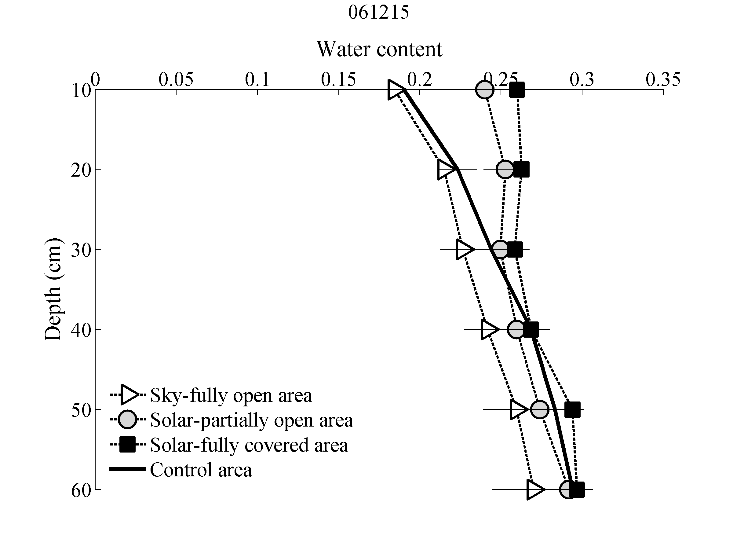 |
| 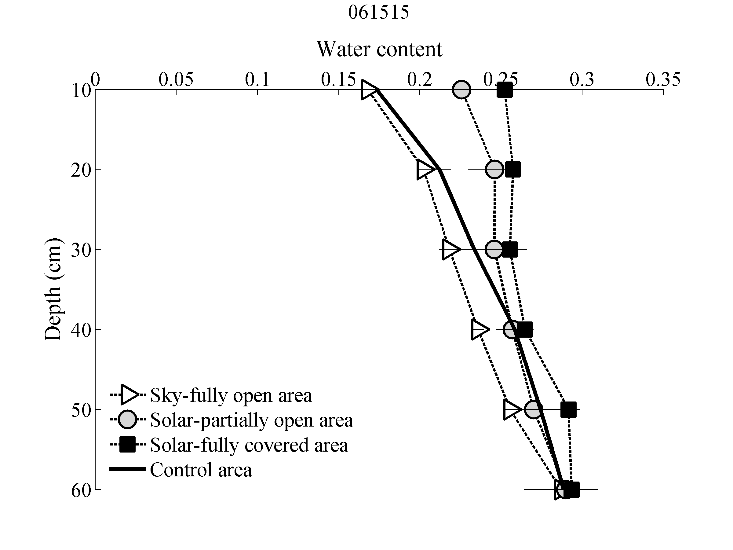 | 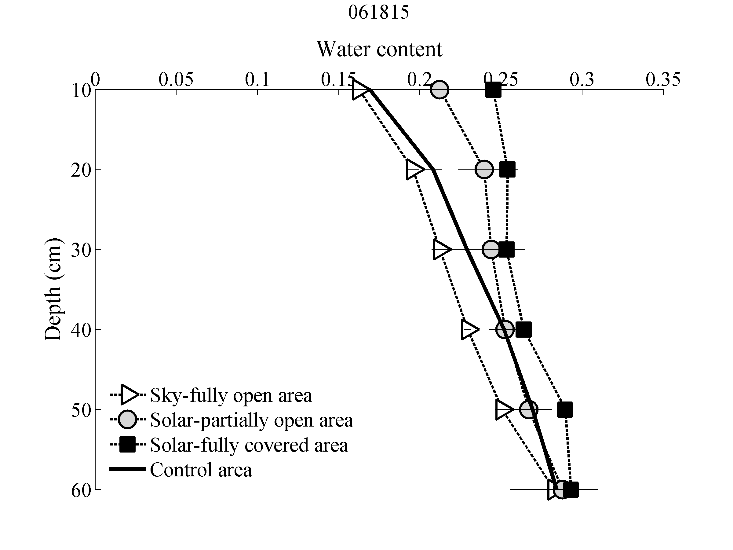 |
| 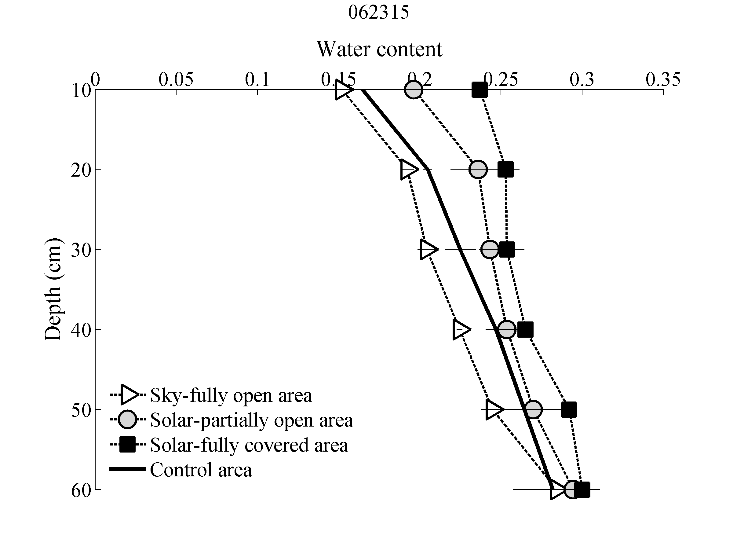 | 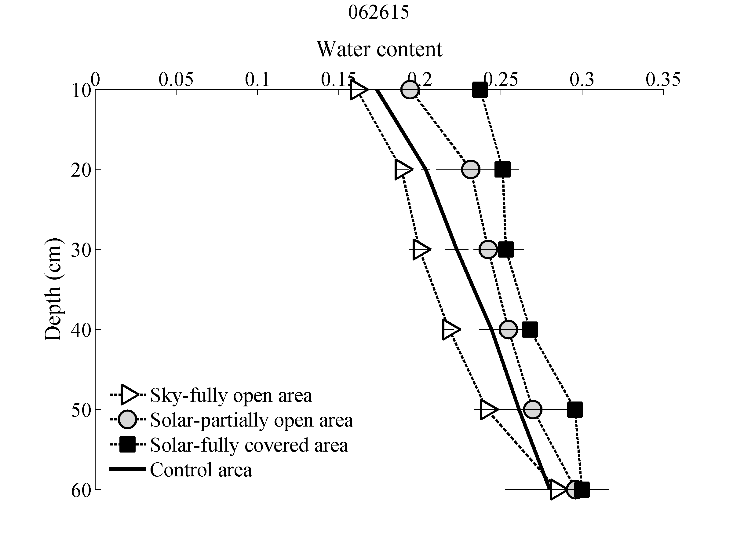 |
| 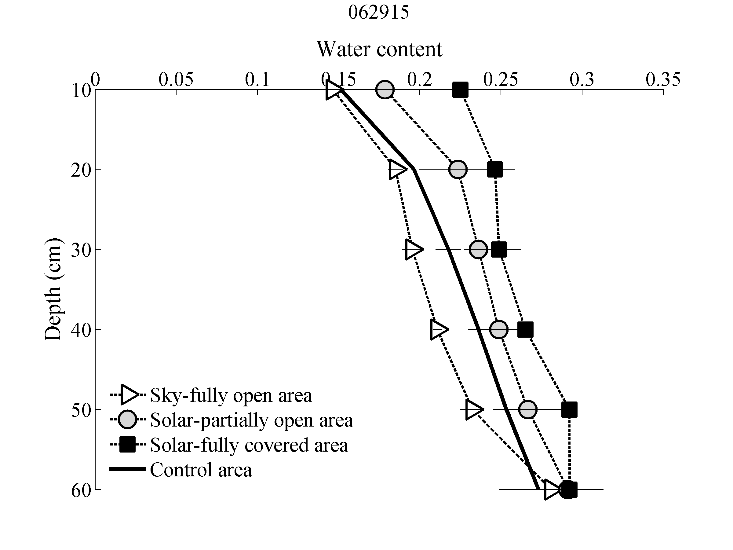 | 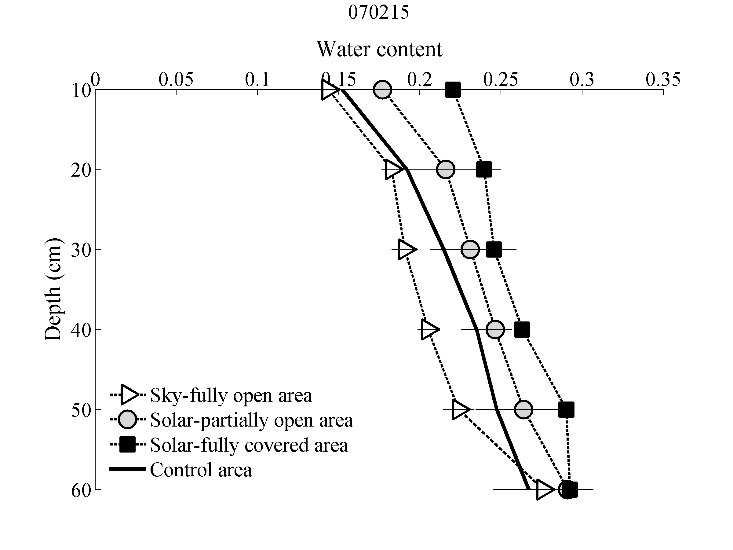 |
| 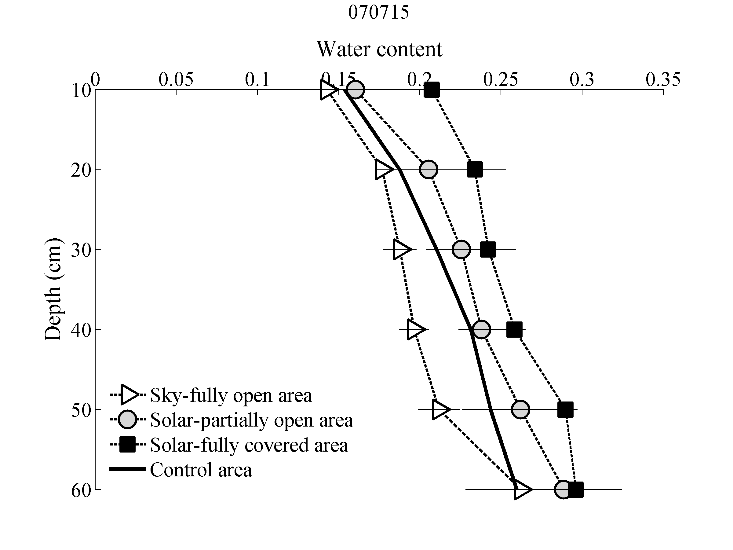 | 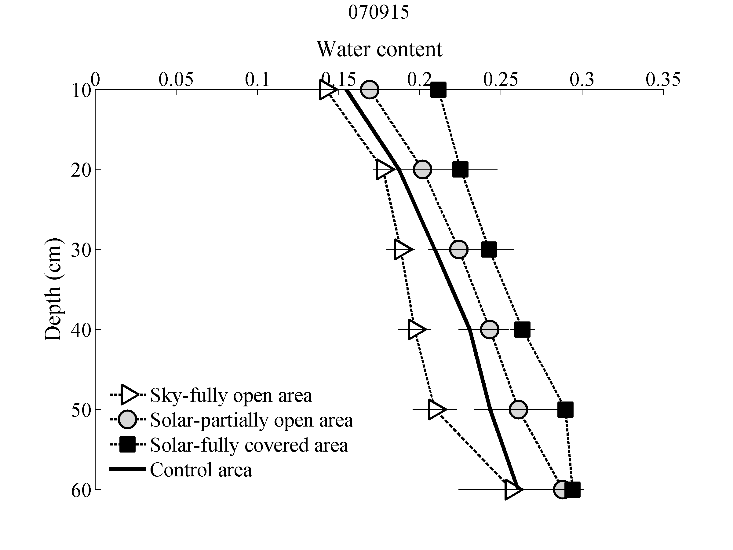 |
| 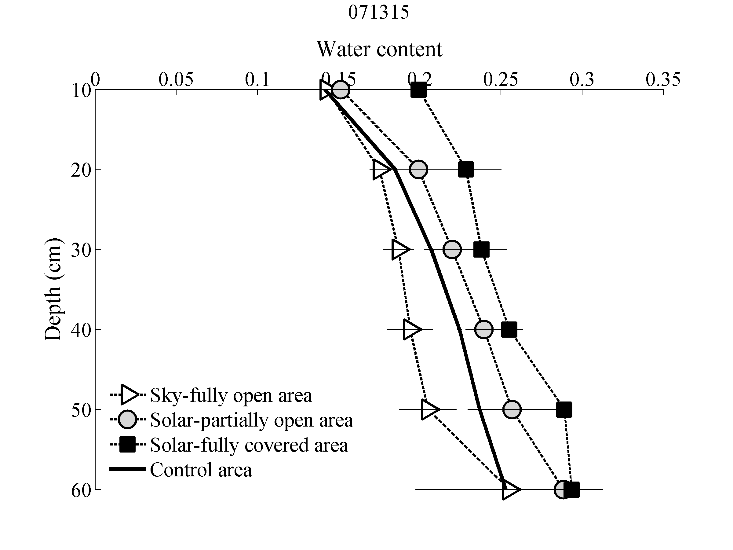 | 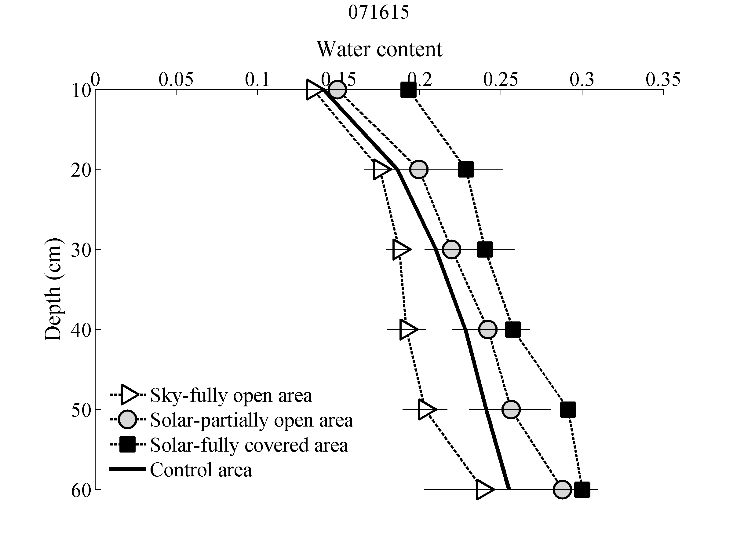 |
| 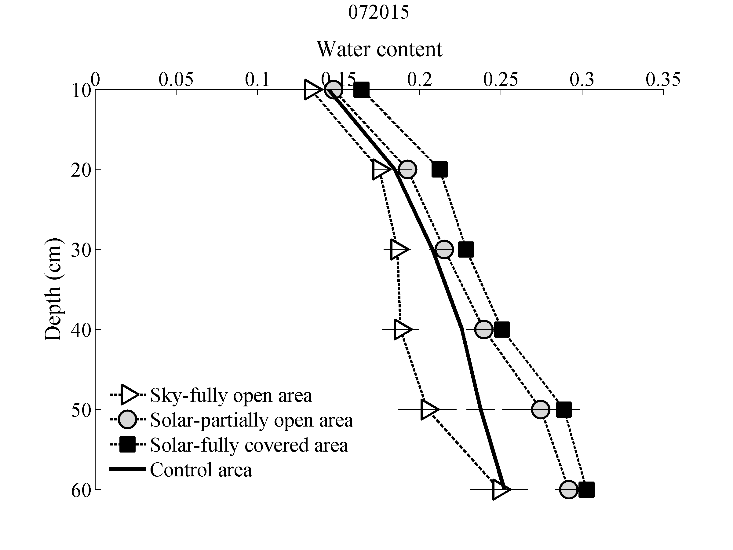 | 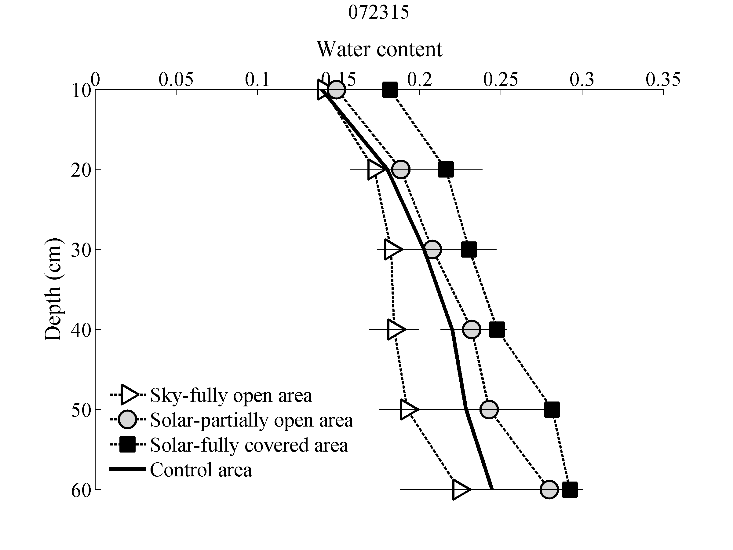 |
| 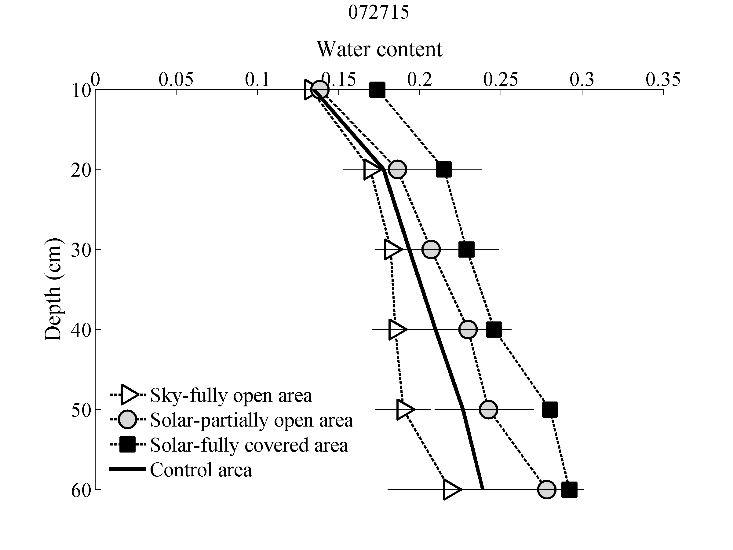 | 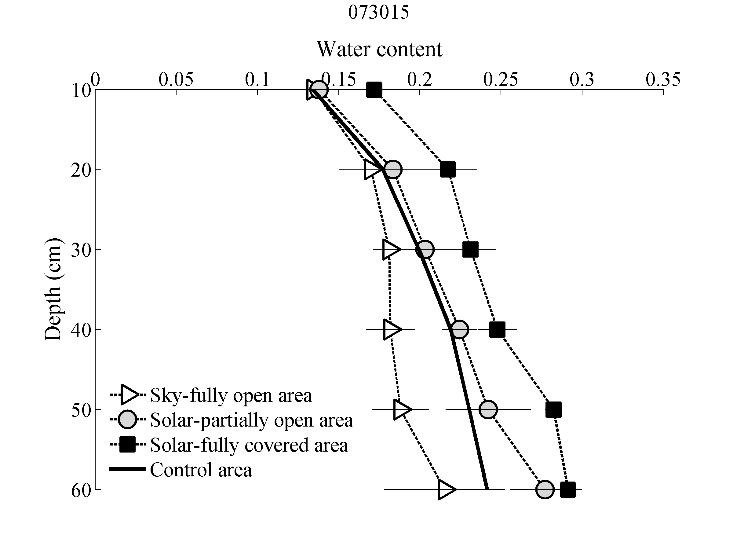 |
| 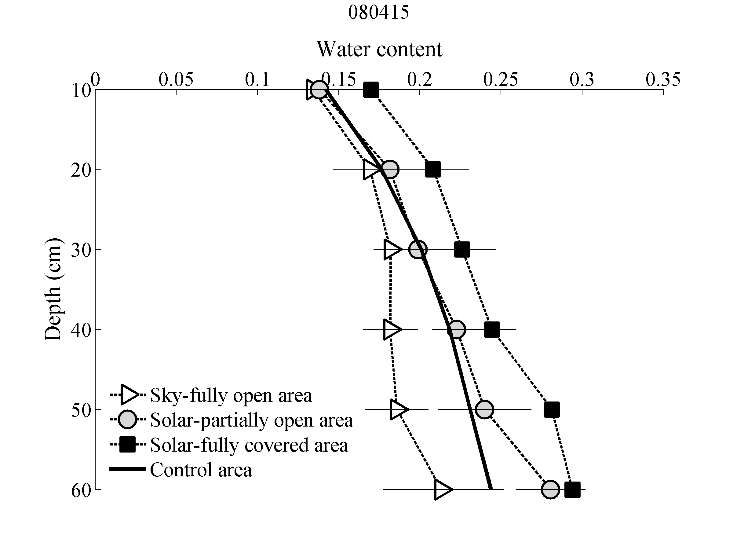 | 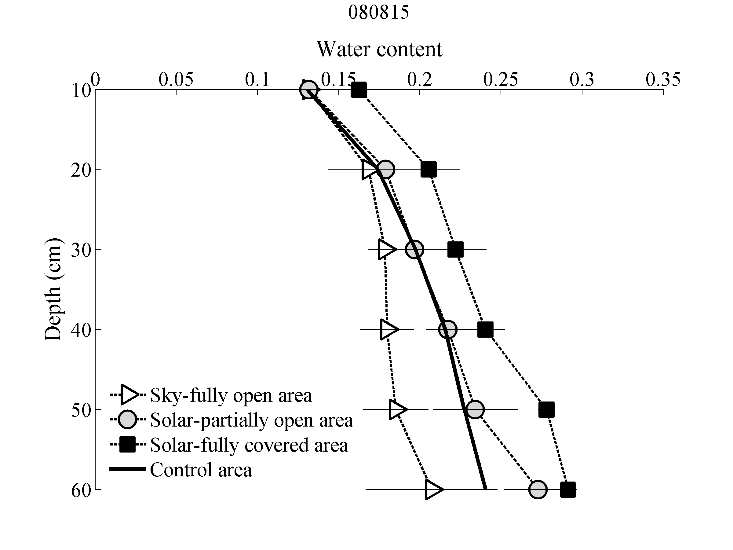 |
| 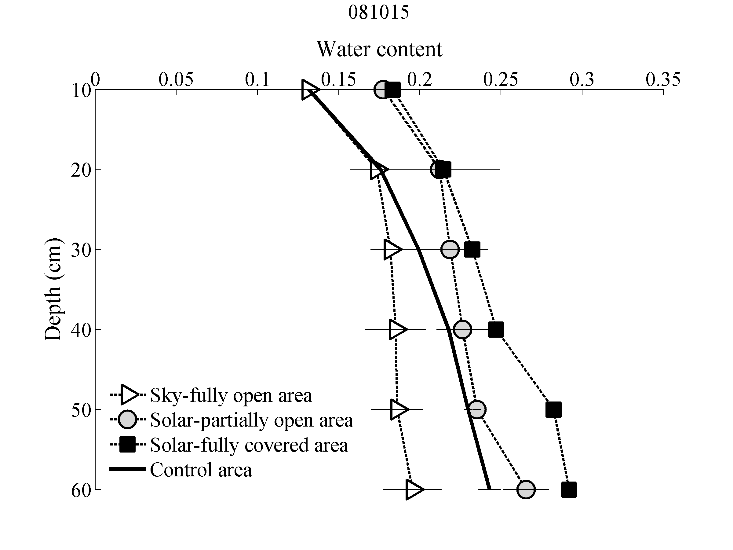 | 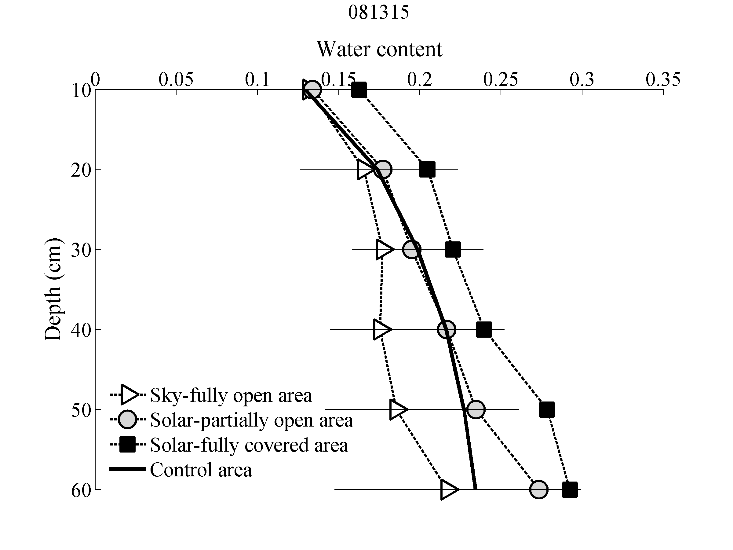 |
| 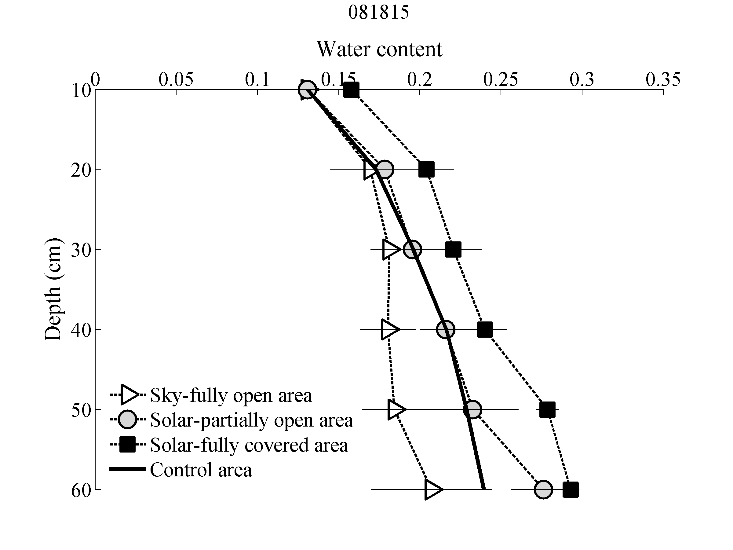 | 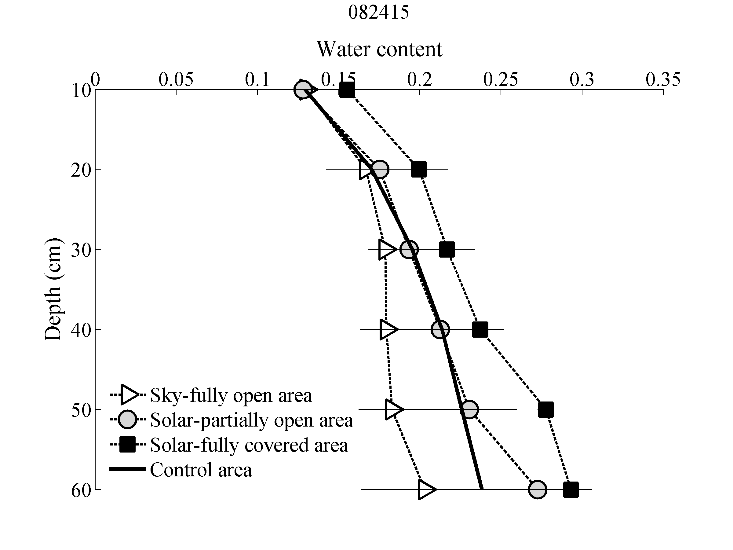 |
| 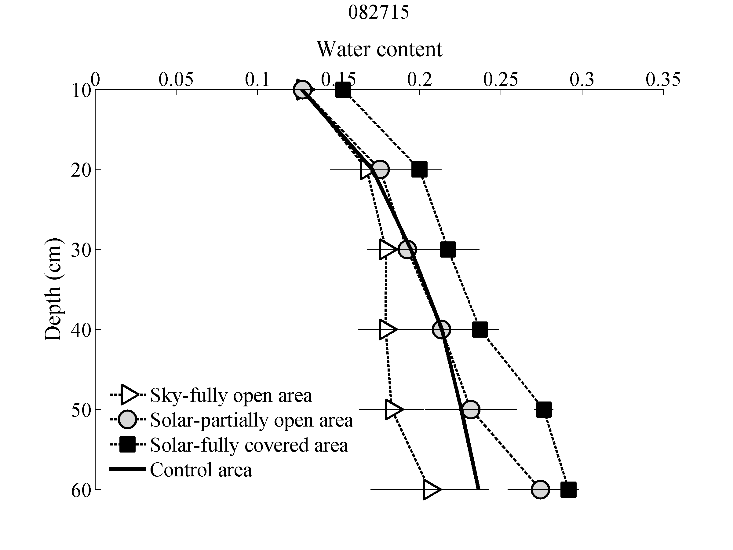 |  |

Supplement: S3 Appendix — Figure A: Selected normalized soil moisture profiles from data sampling to show the change in soil moisture through growing season: May 06–2015 to August 27–2015. The dates are mentioned on top of each figure with mmddyy format. (DOCX) [file pone.0203256.s003.docx]
